# Z‐Scheme Heterojunction Photocatalysts for Energy and Environmental Applications

**DOI:** 10.1002/open.70338

**Published:** 2026-11-12

**Authors:** Mahalakshmi Ramar, Radhakrishnan Kotham, Manikandan Ayyar, Subasree Nattarasu, Zeinebou Yahya, Lalitha Gnanasekaran, Shanmugapriya Dharani, Abinet Gosaye Ayanie

**Affiliations:** ^1^ Department of Chemistry Karpagam Academy of Higher Education Coimbatore Tamil Nadu India; ^2^ Centre for Material Chemistry Karpagam Academy of Higher Education Coimbatore Tamil Nadu India; ^3^ Centre for Advanced Solar Cell Technologies Karpagam Academy of Higher Education Coimbatore Tamil Nadu India; ^4^ Department of Chemistry School of Basic Sciences Vels Institute of Science, Technology and Advanced Studies (VISTAS) Chennai Tamil Nadu India; ^5^ Department of Physics College of Science Qassim University Buraidah Al‐Qassim Saudi Arabia; ^6^ International Center of Nanotechnology and Functional Materials Instituto de Alta Investigación, Universidad de Tarapacá Arica Chile; ^7^ Vicerrectoría de Investigación y Postgrado Universidad de La Serena La Serena Chile; ^8^ Department of Mechanical Engineering Adama Science and Technology University Adama Ethiopia

**Keywords:** interfacial charge transfer, solar energy conversion, sustainable consumption, waste management, Z‐scheme heterojunctions

## Abstract

Z‐scheme heterojunction photocatalysts offer a promising strategy for achieving strong oxidation–reduction potentials while enhancing the spatial separation of photogenerated charge carriers for solar‐driven chemical transformations. This review develops a comprehensive framework connecting interfacial structure, charge transfer dynamics, mechanistic evidence, catalytic performance, stability, and scalability. Type‐II, mediator‐based, all‐solid‐state, and direct Z‐scheme systems are critically compared with emerging S‐scheme architectures, emphasizing band alignment, Fermi‐level equilibration, interfacial band bending, built‐in electric fields, defects, morphology, surface states, and interface chemistry. Mechanistic interpretations are evaluated using complementary techniques, including Mott–Schottky analysis, valence‐band XPS, Kelvin probe force microscopy, surface photovoltage spectroscopy, in situ/operando XPS, electron paramagnetic resonance, transient and ultrafast spectroscopy, isotope labeling, and DFT calculations. Advanced interface engineering strategies involving doping, vacancy regulation, dimensional control, conductive mediators, cocatalysts, and hierarchical architectures are discussed for water splitting, hydrogen evolution, H_2_O_2_ generation, CO_2_ conversion, and environmental remediation. Key challenges include mechanistic ambiguity, inconsistent performance evaluation, photocorrosion, elemental or mediator leaching, interfacial degradation, catalyst recovery, real wastewater treatment, and long‐term stability. Future directions emphasize standardized testing, immobilization, reactor engineering, waste management, resource recovery, techno‐economic assessment, and life‐cycle analysis to enable scalable, low‐cost, and sustainable solar energy and environmental technologies for practical deployment across diverse operating conditions and real‐world industrial applications at scale.

## Graphical abstract

This review provides a comprehensive framework for understanding Z‐scheme heterojunction photocatalysts, linking interface engineering, charge transfer mechanisms, characterization strategies, and catalytic performance. It highlights applications in water splitting, CO_2_ conversion, H_2_O_2_ generation, and environmental remediation while identifying challenges in mechanistic verification, stability, standardization, scalability, and life‐cycle assessment toward sustainable solar‐driven technologies.

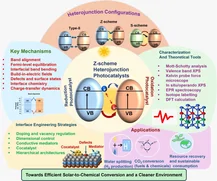

## 1 Introduction

A new junction in the application of photocatalysis in energy and environmental applications is the use of Z‐scheme heterojunction (ZSHJ) photocatalysts, which has both high redox potential and efficient charge separation. Graphitic carbon nitride (g‐C_3_N_4_, gCN) and its derivatives are widely investigated among semiconductors; they have chemical stability, tunable electronic structure, and charge carrier separation, making them suitable component for direct ZSHJs in water splitting, CO_2_ reduction, and pollutant degradation. However, pure gCN has a low surface area, poor light absorption, and low recombination rates, which complicates the construction and charge transfer regulation of ZSHJs and requires solutions in the development of bandgap engineering, morphology regulation, and scalable green synthesis [1].

Recent studies have addressed these limitations thorough various Z‐scheme architectures. The ZnIn_2_S_4_/NiWO_4_ nanostructure exhibits a hydrogen evolution rate of 16.3 mmol g^−1 ^h^−1^, which is 3.57 times faster than that of ZnIn_2_S_4_, which was attributed to the efficient charge transfer in the Ag_2_MoO_4_/Bi_4_Ti_3_O_12_ heterojunctions and enhanced pollutant degradation, confirmed by both the X‐ray diffraction (XRD) and the photoluminescence (PL) analysis and the electrochemical analysis [2, 3]. Others are Ag_3_VO_4_/BiVO_4_ to reduce Cr^6+^ [4], large π‐delocalized g‐C_3_N_4_/P1Cl‐T polymers with 111.8 mmol^−1^g^−1^ H^2^ [5], and 2D/2D g‐C_3_N_4_/Bi_4_Ti_3_O_12_ with long carrier lifetimes [6]. The composites BiOIO_3_/g‐C_3_N_4_ allow the degradation of trichlorophenol by the use of I_3_^−^/I^−^ reduction pathways [7], and CdS@CoWO_4_ spheres promote the photocatalytic degradation of micropolutants [8].

Ternary and hierarchical systems increase functionality, reduced graphene oxide (rGO)/g‐C_3_N_4_/Fe_2_O_3_ to produce H_2_ [9], MoO_3_/g‐C_3_N_4_ to produce H_2_, 87‐fold higher H_2_ evolution rate [10], and Ag_3_PO_4_/g‐C_3_N_4_ to produce O_2_ [11]. Recent heterostructure designs consist of CoS/CdS at carbon nitride double‐shells [12], g‐C_3_N_4_/BiOI/CdS double Z‐schemes [13], ZnO/MoS_2_ nanoarrays based on piezoelectric effects [14], and ZnIn_2_S_4_/TiO_2_ to selectively reduce 4‐nitroaniline [15]. Multinary SnIn_4_S_8_/ZnIn_2_S_4_ has a high H_2_ evolution rate [16], WO_3_/CDots/CdS are all‐solid‐state Z‐schemes with enhanced photostability [17], LaFeO_3_/g‐C_3_N_4_ composites with enhanced dye degradation rate [18], and AgBr/P‐g‐C_3_N_4_ promotes the photocatalytic degradation [19]. Agnosals Cd nanosheets designed as Cu_2_S/Zn_0__.__67_Cd_0__.__33_S exhibit atomic in‐plane Z‐scheme transfer with H_2_ yield of 15.27 mmol^−1^g^−1^ H_2_ [20]. New ternary strategies are CdS/BiOBr/Bi_2_O_2_CO_3_ to degrade herbicides [21], g‐C_3_N_4_/Ag_3_ PO_4_/Ag_2_MoO_4_ to evolve oxygen [22], and Zn_0__.__5_Cd_0__.__5_S/Au@g‐C_3_N_4_ to reduce CO_2_ to methanol [23]. Combined, these developments demonstrate the potential of Z‐scheme photocatalysts in solar energy conversion, environmental remediation, and water purification and indicate the need to design interfaces, optimize light collection, and scale‐up production.

Altogether, ZSHJs are the heterojunction architecture to the effective transformation of solar‐driven redox reactions with large oxidative and reduction potentials. The interface design, morphology control, and heterostructure design that have delivered interface and morphology engineering have yielded improvements in solar energy conversion and environmental remediation. However, there are challenges on the means through which long‐term stability, fabrication on a large scale, and standardized performance evaluation will be achieved. Additional in situ characterization, computational modeling, and data‐driven design activities are necessary to augment mechanistic understanding and accelerate materials discovery. The review aims to essentially critically examine the structural system, charge carrier processes, and mechanistic knowledge on Z‐scheme photocatalysts and how it can be utilized in connecting fundamental research with scalable energy conversion and environmental remediation technologies.

## 2 Fundamentals of Charge Transfer Mechanisms

### 2.1 Type‐II Heterojunctions and Their Transition to Z‐Scheme Architectures

ZSHJs not only increase carrier lifetimes but do not decrease redox potentials in the manner that type‐II heterojunctions do, which not only promotes charge separation but also diminishes oxidation reduction capability. The energy band engineering based on the concept of absolute electronegativity inhibits type‐II alignment and allows Z‐scheme transfer. To give an example, a GaPO_4_/α‐MoC/Ga_2_O_3_ system lacks type‐ii interaction and the Ga_2_O_3_ CB, VB, and Fermi levels are higher than the GaPO_4_, enabling the system to recombine through α‐MoC and fourfold the increase in CO_2_ reduction than Ga_2_O_3_ [24].

Heterostructures 2D heterostructures possess high interfaces and have tunable bandgaps. Z‐scheme behavior of a holey C_1_/h2D‐C_2_ N/C_3_B composite is demonstrated in water splitting, and strain‐modulated band edges have all been found to be highly redox active [25]. Similarly, among 24 van der Waals (vdW) WSe_2_/HfZrCO_3_ heterostructures, a single Z‐scheme was found to have stability, positive charge transfer, and hydrogen generation potential [26]. Z‐schemes can also be used in biomedicine in photocatalysts. Direct FeS_2_/Fe_2_O_3_ Z‐scheme thermally oxidized pyrite nanosheets (FeS_2_ core/Fe_2_O_3_ shell/TOPY NSs) generate ROS and have photothermal and imaging capabilities [27]. Semiconductors, MOFs, and hybrid heterostructures have been studied in type‐II, Z, and S‐systems in environmental remediation by reducing Cr(VI) with the aid of H_2_ generation [28]. The transition metal dichalcogenides can be provided as alternative directions. Janus MoSSe/Ga_2_SSe vdW heterostructures can be tuned to alignments and high intrinsic fields, and one Z‐structure has reported high visible UV absorption and STH efficiencies of 11.84% and 10.13% [29]. Single semiconductors are, however, a compromise between bandgap, light harvesting, and redox power. Type‐II is not as problematic, but since they have lower potentials, Z‐scheme structures are sought [30].

It is the interface control, which is important. Cu_2_O/g‐C_3_N_4_ p‐n heterojunctions with lotus seedpod‐like architecture yielded H_2_ evolution of 637.5 µmol/g^−1^h^−1^. Combined, these publications confirm that the correct placement of band and interfacial design is the main solution to the promotion of Z‐scheme photocatalysts, to overcome the type‐II limitations, to enable the effective solar‐to‐chemical transformation, and to clean the environment, as shown in Table 1.

**TABLE 1:** Summary of representative studies on type‐II heterojunctions and their transition to Z‐scheme architectures.

Photocatalyst system	Mechanism/design strategy	Application/outcome	Reference
GaPO_4_/α‐MoC/Ga_2_O_3_	Avoidance of type‐II alignment via band engineering; α‐MoC mediator enables recombination of GaPO_4_‐CB electrons and Ga_2_O_3_‐VB holes	Fourfold enhancement in CO_2_ photoreduction activity compared to pristine Ga_2_O_3_	[24]
h2D‐C_2_N/C_3_B (2D heterostructure)	DFT‐based strain modulation tunes band edges; preserves strong redox potentials in CB of C_3_B and VB of h_2_D‐C_2_N	Ideal HER and OER potentials achieved, promising for water splitting	[25]
WSe_2_/HfZrCO_2_ (vdW heterostructure)	First‐principles design of 24 configurations; one Z‐scheme with efficient charge separation	Stable photocatalyst for hydrogen generation in neutral and acidic media	[26]
Thermally oxidized pyrite nanosheets (FeS_2_/Fe_2_O_3_)	Direct Z‐scheme plus Fenton reaction; Fe^3+^/Fe^2+^ redox cycling enhances ROS	Glutathione depletion, O_2_ production, ·OH generation; biomedical photonics and imaging	[27]
Semiconductor/MOF and hybrid heterostructures	Type‐II, Z‐scheme, and S‐scheme designs for redox tuning	Cr(VI) reduction coupled with H_2_ generation for wastewater remediation and energy recovery	[28]
Janus MoSSe/Ga_2_SSe (vdW heterostructure)	Built‐in electric field suppresses recombination; direct Z‐scheme band alignment	High STH efficiency (11.84% and 10.13%) for water splitting	[29]
General limitation of type‐II systems	Charge separation achieved, but oxidation/reduction potentials are weakened	Motivation for Z‐scheme photocatalysts	[30]
Cu_2_O/g‐C_3_N_4_ p–n heterojunction	Lotus‐seedpod‐like interface enhances carrier migration	H_2_ evolution rate of 637.5 µmol g^−1 ^h^−1^ (40× higher than pure g‐C_3_N_4_)	[31]
g‐C_3_N_4_/CoN_4_(111) vdW heterostructure	Bandgap tuning from type‐I to type‐II by strain/electric field	Versatile candidate for solar water splitting	[32]
g‐C_3_N_4_/TiO_2_ nanotube composite	Ratio‐dependent transition from type‐II to Z‐scheme	Optimal H_2_ evolution at g‐C_3_N_4_/TiO_2_ ≈ 0.6	[33]

### 2.2 Z‐Scheme Versus S‐Scheme

Heterojunctions with Z‐scheme and S‐scheme configurations are constructed, which can overcome the principal limitation of typical type‐II systems, where the spatial charge separation can be improved but at the expense of strong redox potentials. Although the mechanistic description of the two Z‐scheme and S‐scheme architectures is different, both of them preferentially retain highly reducing electrons and strongly oxidizing holes. The Z‐scheme model emphasizes selective recombination of low‐energy electrons with weakly oxidizing holes, whereas the S‐scheme model additionally describes the interfacial electronic processes responsible for this selective charge transfer, including Fermi‐level equilibration, band bending, and formation of a built‐in electric field (BIEF) [34]. In a direct Z‐scheme, the photogenerated electrons from the oxidation photocatalyst recombine directly with the photogenerated holes of the reduction photocatalyst, leaving the high‐energy electrons and holes available for reduction and oxidation reactions, respectively. A heterojunction comprising 1T/2H‐MoS_2_/ZnS was attributed as a direct Z‐scheme based on interfacial electronic coupling, electron paramagnetic resonance (EPR) analysis, and density functional theory (DFT) calculations. Nevertheless, an increase in photocatalytic activity alone does not confirm a Z‐scheme mechanism; it is necessary also to experimentally verify the postulated direction of charge transfer and retention of the redox‐active carriers [35].

A more rigorous sense of distinction between S‐scheme charge transfer and conventional type‐II is given by the interfacial electronic equilibration. When an interface is formed, the electronic structure and work function mismatches lead to charge rearrangement at the interface. This causes band bending and the formation of an electric field where weakly reducing electrons and weakly oxidizing holes will recombine. Most importantly, these electronic features need to relate to a measurable carrier transfer behavior and not just band alignment. A new donor–acceptor covalent organic framework (D–A COF)/ZnIn_2_S_4_ quantum‐dot heterojunction shows that combining X‐ray photoelectron spectroscopy (XPS) and Kelvin probe force microscopy (KPFM) with EPR works to analyze electronic interactions at heterointerfaces and the corresponding S‐scheme charge transfer route. The prepared heterostructure displayed much enhanced H_2_O_2_ production than the sole components, which indicates that interfacial electronic coupling can facilitate carrier separation while maintaining the redox‐active carriers for O_2_ reduction [NEW REF]. Accordingly, the S‐scheme assignment should be based on concomitant evidence for interfacial charge redistribution, electric field formation, carrier migration, and redox species generation, not just on photocatalytic activity [36]. On the other hand, the S‐scheme mechanism is associated with interfacial electronic equilibration between two semiconductors with different Fermi levels. Upon contact, electron distribution takes place until the Fermi levels are aligned, creating interfacial charge accumulation, band bending, and BIEF. This field will promote the selective recombination of the photogenerated electrons and holes with weaker redox abilities upon illumination while preserving the strongly reducing electrons and strongly oxidizing holes. Consequently, the BIEF and the corresponding band bending offer electronic basis for the carrier selection behavior of the S‐scheme. The proposed S‐scheme charge transfer pathway was supported by photoirradiated Kelvin probe measurements and in situ irradiated XPS (ISIXPS) [37, 38].

The assessment of interfacial electronic structure in Bi_2_MoO_6_/MoS_2_ 2D/2D heterojunctions presents further evidence that KPFM, valence‐band XPS, and Mott–Schottky measurement were jointly utilized to establish the heterojunction electronic structure. The electric field generated at the interface is envisioned to facilitate the directional transfer of charge while ensuring that the two components maintain their respective redox capabilities. The evidence combined provides stronger mechanistic evidence support than asserting that an S‐scheme mechanism arises from merely band positions or photocatalytic activity [39]. Examining the interfacial electric field is important for evaluating S‐scheme behavior. Differences in Fermi‐level alignment in facet‐engineered ZnIn_2_S_4_/BiOBr heterojunctions resulted from changing BIEF strength and band bending. The stronger interfacial field enhanced selective recombination of weakly reducing electrons and weakly oxidizing holes, which halted recombination of the conduction‐band electrons of ZnIn_2_S_4_ and strongly oxidizing valence‐band holes of BiOBr. This shows that interface configuration and electronic characteristics can control the S‐scheme charge transfer pathway [40]. The linear dependence of the photocurrent on the laser intensity evidenced two‐photon absorption (TPA) events. KPFM provides 3D images of changes in surface potential, while the in situ irradiated XPS can show illumination‐induced changes in electronic density. Femtosecond transient absorption spectroscopy (fs‐TAS) and EPR give complementary information about carrier transfer kinetics and reactive species. Recent KPFM measurements that were spatially resolved show that the surface potential responses on the reduction and oxidation photocatalysts in S‐scheme systems were different when illuminated [41].

The existence of an interfacial electronic bridge can make it difficult to differentiate the Z‐scheme from S‐scheme. In a g‐C_3_N_4_/V_2_C/ZnIn_2_S_4_ heterojunction, V_2_C MXene functioned as an interfacial charge transfer material, while in situ EPR, fs‐TAS, and DFT computations were used to decipher the electron transfer route. The dynamic measurement is useful because it provides direct evidence of carrier migration (or not) which cannot be resolved from the static band diagrams that were used to propose the heterojunctions mechanisms [42]. Measurements of work function provide another important criterion for determining interfacial charge transfer direction. In TiO_2_/MXene systems, KPFM measurements revealed that the charge transfer direction was determined by the work function difference across the components. By altering the MXene surface, the work function was modified and thus interfacial carrier transfer behavior changed. These findings highlight the need for experimental determination of the work functions when proposing S‐scheme mechanisms instead of inferring solely from the electron transfer direction based on nominal band positions [43].

### 2.3 Mediator‐Based, All‐Solid‐State, and Direct Z‐Scheme Architectures

Effective Z‐scheme photocatalysts rely on interface engineering, and mediator‐based, all‐solid‐state, and direct designs have enhanced charge separation, carrier lifetime, and redox capacity. Conductive components are utilized in mediator‐based systems as electron shuttles. Oxidized TiO_2_/MgIn_2_S_4_ (MIS) composites were prepared, with the MIS microflowers anchored on the Ti_3_C_2_ nanosheets, promoting ciprofloxacin and liberating H_2_ at 92 and 520.3 µmol h^−1^, respectively, depending on the charge transfer by Ti_3_C_2_ [44]. Under an rGO‐mediated CdS/rGO/WO_3_ heterojunction, enhanced charge transport through rGO resulted in 11.69 mmol g^−1 ^h^−1^ (2.3× pure CdS) [45]. MXene‐based electron shuttling was also applied to another system, in which TiO_2_/Ti_3_C_2_TiC/CZUNH was used to produce H_2_O_2_ and H_2_ [46]. The chemically bonded designs also exhibited enhanced photocatalytic performance, a Bi/BiOBr@Bi‐MOF heterojunction was employed to degrade chlortetracycline (97.1% oxygen vacancy‐mediated) using Bi mediation [47], and *N*‐rGO‐mediated NiFe LDH/g‐C_3_N_4_ system exhibited high H_2_ evolution [48].

All‐solid‐state Z‐schemes are redox neutral, that is, they are stable and do not disrupt the environment. A 1T/2H‐MoS_2_@BCN hybrid has reported 290 µmol h^−1^ and 95% degradation of TCH, which compares well with 1T‐MoS_2_ [49]. A NiTiO_3_/Cd_0.5_Zn_0.5_S direct heterostructure generated 1058 µmol h^−1^ of H_2_ and AQY of 34 at 420 nm, without noble metal cocatalysts [50]. Others are Fe_2_O_3_/g‐C_3_N_4_ to reduce CO_2_, carbon dots/WO_3_ to degrade RhB [51], and red P/RGO/WO_3_ to split water (287 mmol g^−1^h^−1^) [52]. Ternary systems also make it more efficient. Bi_4_O_5_I_2_/GO/Bi_2_Sn_2_O_7_ reduced tetracycline by 90.31%, and GO aided in the transfer of charges [53]. Direct Z‐schemes are also promising: MnIn_2_S_4_/g‐C_3_N_4_ nanocomposites were more effective in wastewater removal and H_2_ production [54]; g‐C_3_N_4_/WO_3_ host–guest structures had atomically defined interfaces and could produce H_2_ by using internal electric fields [55]; and CdS@ZnIn_2_S_4_ hollow structures produced stoichiometric H_2_ and H_2_O_2_ [56]. Structural engineering further increases the efficiency of remediation, with g‐C_3_N_4_/WO_3_ homogeneous microspheres having 5.7‐fold greater tetracycline degradation and CuTPP/HBVO vacant oxygen cases demonstrating 4.5‐fold Z‐scheme transfer and effective 2‐chlorophenol degradation at the Cu I active site [57, 58].

A combination of mediator‐based, all‐solid‐state, and direct Z‐schemes is an example of the success of rational interface engineering in energy conversion and environmental remediation. The representative mechanisms are shown in Figure 1a, where the direct ZSHJ (TiO_2_/g‐C_3_N_4_) with charge transfer is shown, where electrons in the TiO_2_ CB and holes in the g‐C_3_N_4_ VB recombine with each other, and where the other charge carriers generate redox reactions to enhance photocatalytic reactions [59]; (b) the schematic of a Z‐scheme system based on redox mediators (WO_3_/Cu_2_O), where the photocatalysts are linked together by redox mediators that drive electron flow between them, facilitating charge separation and producing reactive oxygen species (ROS) for photocatalytic reactions [60]; (c) directed charge transfer under visible light using an all‐solid‐state ZSHJ (g‐C_3_N_4_/MOC‐Q2), where a solid electron mediator enables efficient H_2_ evolution through enhanced redox reactions [61]; and (d) direct Z‐scheme charge transfer in the band structure of p‐BiOCl/*n*‐Bi_2_WO_6_, conceptualizing strong redox potential and efficient photocatalytic performance [62].

**FIGURE 1:**
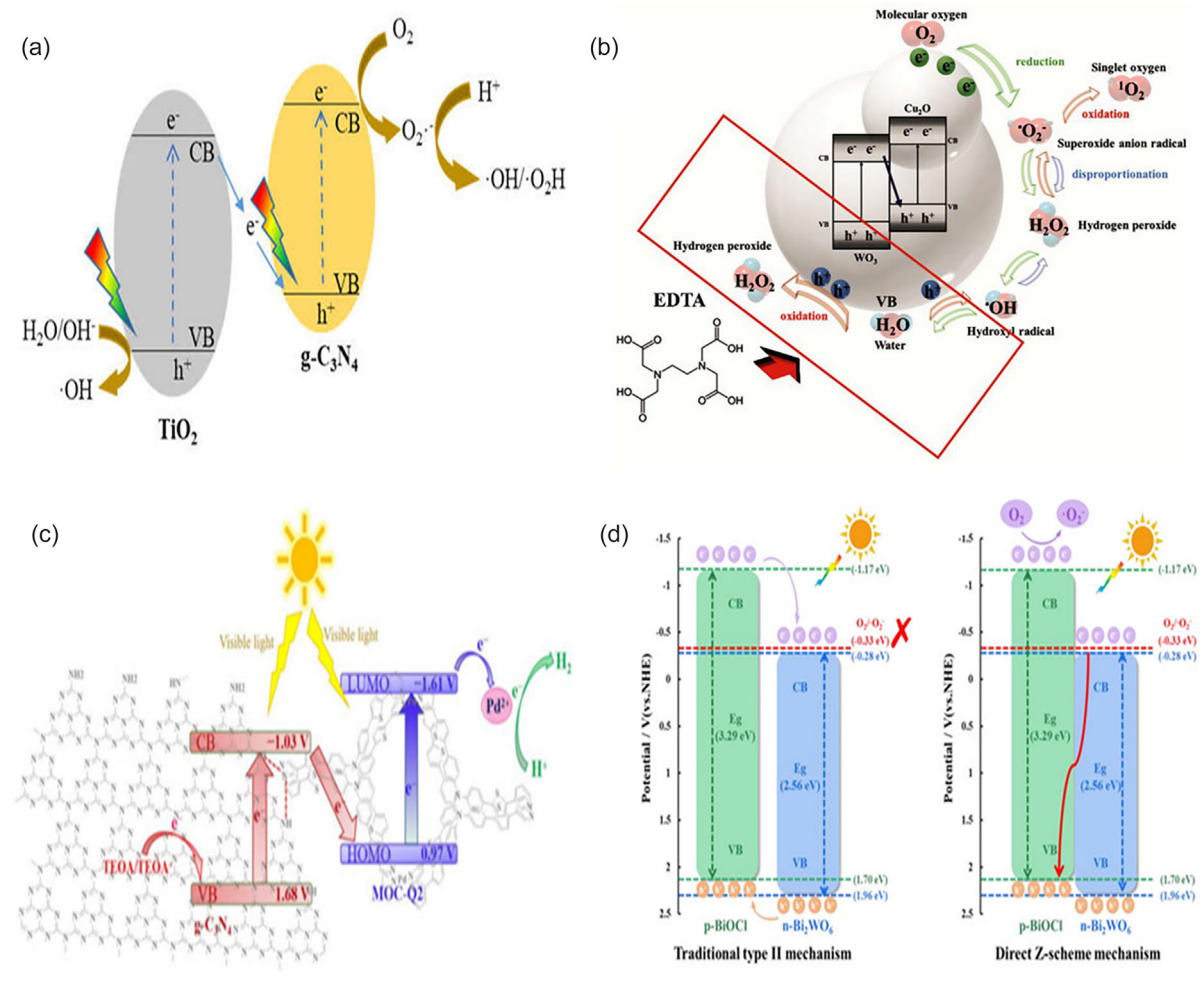
(a) Direct ZSHJ (TiO_2_/g‐C_3_N_4_) with charge transfer, electrons in TiO_2_ CB recombine with holes in g‐C_3_N_4_ VB, and the remaining charge carriers generate redox reactions to boost photocatalysis [59]. (b) Schematic of a mediator‐based Z‐scheme system (WO_3_Cu_2_O with EDTA) in which the photocatalysts are linked together by redox mediators that drive the flow of electrons between them, facilitating the process of charge separation and producing ROS to be used in photocatalytic reactions [60]. (c) Directed charge transfer under visible light using all‐solid‐state ZSHJ (g‐C_3_ N_4_/MOC‐Q2) in which a solid electron mediator facilitates the reaction, leading to efficient H_2_ evolution in response to improved redox reactions [61]. (d) Direct Z‐scheme charge transfers in band structure of p‐BiOCl/*n*‐Bi_2_WO_6_ conceptualizing strong redox potential and efficient photocatalytic performance [62].

### 2.4 Criteria for Identifying and Distinguishing Mechanisms

Type‐II, Z‐scheme, and S‐scheme heterojunctions cannot be distinguished solely on the basis of the proposed band‐alignment diagram but need other experimental evidence. While these mechanisms can promote spatial separation of the photogenerated electron–hole pairs, they have fundamentally different charge transfer pathways and retained redox potentials. So, band structure analysis, charge transfer at the interface, BIEF activation, light‐induced charge transfer, and charge carrier dynamics evaluation must be used for identifying the mechanism. According to recent studies, complementary spectroscopic, photoelectrochemical (PEC), and theoretical approaches are needed to establish reliable charge transfer pathways [63].

The semiconductor type and band‐edge positions are basic criteria for evaluating a proposed heterojunction mechanism. Analysis via Mott–Schottky can determine the semiconductor type and flat band potential, while valence‐band XPS gives the valence‐band maximum. Taking into account the optical bandgap, these assessments also allow for estimating the conduction‐band position and evaluating the thermodynamic feasibility of the expected charge transfer process. The combination of EPR, valence‐band X‐ray photoelectron spectroscopy (VB‐XPS), and Mott–Schottky measurements in the CdIn_2_S_4_/ZnO/PAN system endorsed the suggested interfacial charge transfer pathway, highlighting the importance of relating electronic structure measurements with photocatalytic behavior [64]. When two semiconductors with different electronic properties are brought into contact, this can lead to interfacial electron redistribution, which causes band bending and a BIEF. In S‐scheme heterojunctions, the interfacial field that can be formed can provide directional driving forces for photogenerated carriers. Importantly, KPFM and surface photovoltage measurements can directly probe changes in surface potential associated with charge redistribution. Moreover, through quantitative investigation of BIEF intensity, it has been established that the interfacial electric field is associated with photocatalytic charge utilization. Therefore, BIEF should be evaluated as an experimentally measurable electronic parameter of heterojunction models [65]. The BIEF's relevance can be showcased further using KPFM measurements, both in dark and illuminated conditions. Surface potential measurements of *n*‐type and p‐type semiconductor components show opposite results indicating different surface charge response and interfacial electric field formation. Since they provide experimental information about the charge redistribution direction and how the interface impacts the separation of photogenerated carriers, such measurements can enhance band alignment studies [66].

XPS offers an essential means of identifying the interfacial redistributions of electrons occurring through the shifts of core‐level binding energies. When there is an increase in binding energy, it means the electron density around the element decreases and likewise the reverse. Consequently, the comparison of the individual components and heterojunction in dark conditions can unveil charge redistribution direction after interface formation. ISIXPS can reveal illumination‐induced changes and thus more directly determine the direction of photogenerated electron migration. Evidence of interfacial electron redistribution and light‐induced electron transfer in the COF‐5/ZnIn_2_S_4_ system, inferred from dark‐ and light‐state binding energy shifts, supported the proposed S‐scheme mechanism [67]. It has been highlighted that the capability of a proposed heterojunction to maintain strong redox potentials can serve as an important criterion to distinguish type‐II from Z‐scheme and S‐scheme mechanisms. In a standard type‐II scenario, photogenerated electrons are attracted toward the semiconductor possessing the lower conduction‐band position, while holes migrate toward the semiconductor with the higher valence‐band position. While this pathway can spatially decouple carriers, it can simultaneously weaken the reducing and oxidizing power of the entire component. On the contrary, electrons with higher reduction potential and holes with higher oxidation potential are preferentially preserved in S‐scheme and Z‐scheme mechanisms. Thus, detection of ROS or other radical intermediates by EPR, when combined with band positions obtained experimentally, can provide mechanistic evidence. The heterostructure F‐TiO_2_/CdS can produce more radicals than pure TiO_2_. This means that it can retain the strong reduction capability of CdS and the oxidation capability of F‐TiO_2_. Thus, the authors believe that the S‐scheme charge transfer model proposed also holds true for this heterostructure [68]. An increase in photocurrent response, a decrease in charge transfer resistance, surface photovoltage variations, and an improvement in carrier lifetime can serve as additional evidence for effective interfacial charge separation. For instance, the MoS_2_/Bi_2_S_3_/Bi_2_WO_6_ photoanode showed markedly enhanced photocurrent compared to pristine Bi_2_WO_6_, while the combined structure and electronic analyses were employed to rationalize its improved photoelectrochemical performance using a coupled heterojunction charge transfer pathway. When considered with band positions and interfaces as opposed to independently assigning any one mechanism, such evidence is particularly useful [69].

Static band structure measurements cannot fully determine the actual pathway of photogenerated carriers. Direct study of charge carrier dynamics provides stronger mechanistic evidence. fs‐TAS resolves ultrafast carrier transfer processes, in situ irradiated XPS tracks illumination‐induced changes in electronic density, and KPFM measures surface potential variations. There is increasing interest in using these techniques to distinguish S‐scheme charge transfer from typical type‐II behavior [70]. By changing carrier concentration, Fermi‐level alignment, surface states, and the strength of the BIEF, the interfacial charge transfer behavior can be altered significantly through defect engineering. It was demonstrated in ZnIn_2_S_4−*x*_/MoO_3−*x*_ that simultaneous oxygen‐ (O‐) and sulfur‐ (S‐) vacancy engineering can alter the interfacial electric field and charge transfer dynamics while improving photocatalytic hydrogen evolution. Observations, such as these imply that defect‐triggered electronic structure modifications, could potentially supplement our understanding of defect‐induced carrier separation enhancement origin, although defect effects should be disentangled from the intrinsic heterojunction mechanism [71]. In the end, a robust mechanistic assignment should result from the convergence of various independent observations and not from a single characterization technique. The thermodynamic framework is established by band positions; the electronic structure is defined by Mott–Schottky and VB‐XPS measurements; interfacial and light‐induced electron redistribution is established by XPS and in situ irradiated XPS; evidence of surface potential changes and BIEF formation is provided by KPFM/surface photovoltage (SPV); the changes in reactive species and redox behavior are verified by EPR; and separation of the carrier migration. The interfacial interactions’ role in charge separation has been further demonstrated in a TiO_2_/ZnIn_2_S_4_ S‐scheme system through in situ XPS, in situ KPFM, and DFT analyses [72]. The summary experimental and theoretical criteria for distinguishing type‐II, Z‐scheme, and S‐scheme heterojunctions are shown in Table 2.

**TABLE 2:** Experimental and theoretical criteria for distinguishing type‐II, Z‐scheme, and S‐scheme heterojunctions.

Characterization technique	Key information obtained	Mechanistic significance	Evidence strength	Major limitation	Reference
Mott–Schottky analysis	Semiconductor type, flat band potential and approximate CB position	Establishes the basic band structure and thermodynamic feasibility of charge transfer	Moderate	Does not directly reveal the direction of photoinduced charge migration	[63]
VB‐XPS	Valence‐band maximum and VB electronic structure	Determines the oxidation‐potential framework required for evaluating possible charge transfer pathways	Moderate	Provides mainly static information and cannot independently distinguish Z‐scheme from S‐scheme	[64]
KPFM/SPV	Surface potential distribution, work function variation, and light‐induced potential changes	Provides evidence for interfacial charge redistribution and BIEF formation	Strong	Surface potential can be influenced by defects, adsorbates, and environmental conditions	[65]
Dark/light KPFM measurements	Potential changes before and after illumination	Helps determine light‐induced carrier redistribution and the influence of the interfacial electric field	Strong	Requires careful interpretation of potential polarity and surface charging	[66]
Dark‐state XPS	Core‐level binding energy shifts after heterojunction formation	Indicates equilibrium electron redistribution between the two components	Moderate–strong	Binding energy shifts can also originate from chemical state changes, defects or charging	[67]
EPR/ESR and radical trapping experiments	Formation of •O_2_^−^, •OH and other reactive species	Indicates whether the surviving carriers possess sufficiently strong reduction/oxidation potentials	Moderate	Reactive species detection does not directly establish the carrier transfer direction	[68]
Photocurrent and EIS	Carrier separation/extraction and interfacial charge transfer resistance	Supports improved carrier separation and transport after heterojunction formation	Weak–moderate	Cannot independently distinguish type‐II, Z‐scheme and S‐scheme pathways	[69]
fs‐TAS	Ultrafast carrier transfer, accumulation, and recombination dynamics	Provides dynamic evidence for interfacial charge transfer processes and their timescales	Strong	Complex transient signals require careful spectral assignment	[70]
Defect/vacancy engineering with electronic characterization	Changes in carrier concentration, surface states, Fermi‐level alignment, and interfacial electronic structure	Helps determine how defects modify charge redistribution and BIEF‐assisted carrier dynamics	Moderate	Defect effects may be independent of the intrinsic heterojunction mechanism	[71]
Combined in situ XPS + KPFM + DFT	Experimental charge redistribution, surface potential changes, and theoretical electronic structure	Provides convergent evidence for interfacial charge transfer and proposed S‐scheme behavior	Strong	Requires multiple complementary measurements and consistent interpretation	[72]

## 3 Design Principles of Z‐Scheme Photocatalysts

### 3.1 Band Alignment and Fermi‐Level Engineering

ZSHJs have been shown to be charge separated, transferred, and photocatalyzed by the energy band structure. The ability of heterojunctions to store redox potential and to control carrier migration can be realized by precise band alignment and tuning of the Fermi level using doping, interface modulation, vacancy engineering, and mediator selection schemes. The production of sulfur vacancies and red phosphorus (RP) resulted in a ZnIn_2_S_4_‐S/carbon nanotubes (CNTs)/RP nanocomposite capable of evolving H_2_ at 1639.9 µmol g^−1^h^−1^ because it was high in Fermi‐level difference and could transfer it through the Z‐scheme [73]. Heterojunctions of ZnS/SnS_2_ dissolved 93.46% of tetracycline, and Z‐scheme overlaps were checked by band offsets (ΔECB = 1.02 eV; ΔEVB = 0.22 eV) [74]. Nitrogen doping as well as CdS deposition on ZnIn_2_S_4_ gave CdS/*N*‐ZIS heterostructures with high interfacial fields, producing 15.7 µmol g^−1 ^h^−1^ H_2_ (7.4× pristine ZIS) [75]. The type‐I, g‐ZnO vdW heterostructures were studied through the DFTs, and a potential difference of 11.18 eV was reported, indicating that external strain and electric fields could facilitate transitions between type‐I, type‐Z, and type‐II modes [76]. Facet engineering of directional charge transfer was also reported in TiO_2_‐Pd‐Cu_2_O, which confirmed the Fermi‐level sequence (TiO_2 _< Pd < Cu_2_O) to facilitate degradation of phenols [77]. Fermi level changes in BiVOx/BiOBrOv changed the type‐II transfer to a Z‐schemed mechanism and reached 91% of oxytetracycline degradation [78].

The BiOI/Pt/g‐C_3_N_4_ system degraded phenols 6.0 times better than bare g‐C_2_N_4,_ and the catalyst Pt mediated efficient charge transfer [79]. A WO_3_/Cu/g‐C_3_N_4_ heterojunction was used to maximize the generation of •OH, with the intermediate Fermi level of Cu, which was found to degrade 4‐nonylphenol 11.6× faster than WO_3_/g‐C_3_N_4_ [80]. An ultrathin ZnIn_2_S_4_/g‐C_3_N_4_ had high interfacial fields and high H_2_ evolution without cocatalysts [81]. However, vdW heterojunctions of ZnTe/ZrS_2_ displayed strain‐induced light absorption, type‐II alignment, and 14.3% conversion efficiency [82]. These results indicate that directional charge transfer is indeed controllable accurately by doping, vacancies, facet control, and choice of mediators, enhancing the alignment of bands and positioning of bands at the Fermi level to maintain redox potential and high photocatalytic activity in the generation of solar fuels and environmental remediation.

An example of Fermi‐level and band alignment engineering in Z‐schemes is shown in Figure 2a TiO_2_/Bi_2_O_3_ heterojunction band alignment that illustrates engineered Fermi‐level alignment, direct Z‐scheme charge transfer and enhanced redox capabilities, and photocatalytic activity under visible light [83]; (b) WO_3_/Co_2_SnO_4_ heterojunction band structure that demonstrates optimal Fermi‐level alignment, direct Z‐scheme charge transfer, and improved redox performance under visible light [84]; (c) charge transfer, internal electric field formation in a BOC/BOI ZSHJ, catalyzing redox reactions, even without irradiation [85]; and (d) ZSHJ band alignment with engineered Fermi‐level positioning and free energy profile to accelerate H_2_ evolution [86].

**FIGURE 2:**
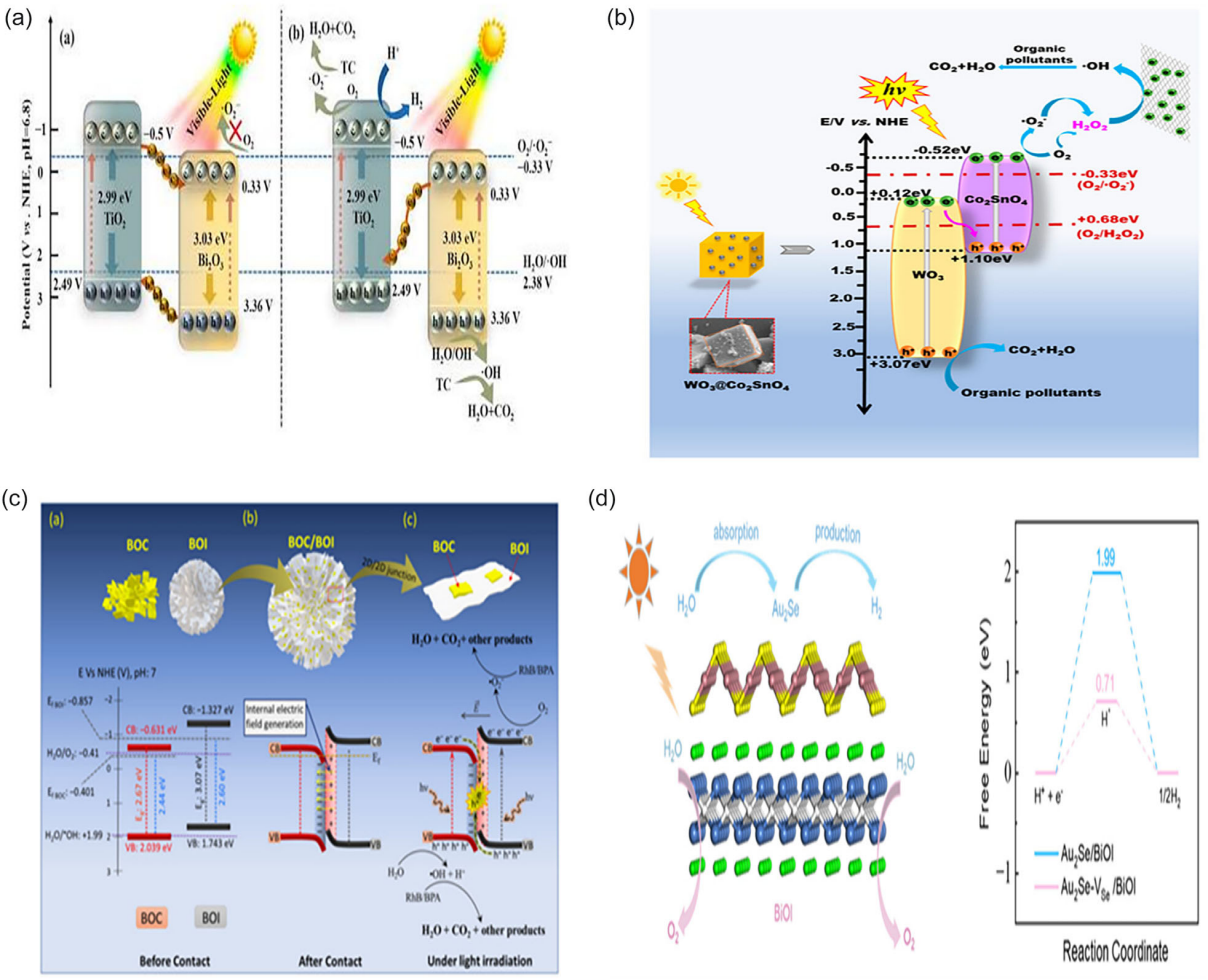
(a) A TiO_2_/Bi_2_O_3_ heterojunction band alignment that demonstrates how the Fermi level can be engineered to enhance direct Z‐scheme charge transfer and increased redox capacity and photocatalytic activity under visible‐light irradiation [83]. (b) A WO_3_ at Co_2_SnO_4_ heterojunction band structure illustrating the best alignment of the bands and Fermi level engineering of such that can efficiently transfer charge through direct Z‐schemes to pollute materials, which react strongly to visible‐light irradiation [84]. (c) Charge transfer and internal electric field formation in a ZSHJ of BOC/BOI in the absence of light irradiation along with an efficient redox reaction [85]. (d) ZSHJ with charge transfer band alignment, H_2_ evolution facilitated by Fermi‐level engineering, and the corresponding free energy profile [86].

### 3.2 Built‐in Electric Fields and Interfacial Band Bending

The formation of a BIEF at a semiconductor interface is a fundamental consequence of interfacial charge redistribution and plays a central role in directing photogenerated carrier transport. When two semiconductors with different electronic structures are brought into contact, differences in Fermi levels drive electron redistribution until interfacial equilibrium is established. The resulting space charge region, band bending, and internal electric field modify the local energy landscape and provide a driving force for directional carrier separation. In S‐scheme heterojunctions, this interfacial electric field is proposed to selectively drive the migration and recombination of photogenerated carriers with weaker redox abilities while preserving the strongly reducing electrons and strongly oxidizing holes. Spatially resolved KPFM measurements have directly visualized illumination‐induced surface potential changes and carrier accumulation on the reduction and oxidation photocatalysts, providing experimental evidence for the role of the interfacial electric field in S‐scheme charge separation [87].

The BIEF is closely associated with interfacial band bending, which originates from charge redistribution within the space charge region. Rather than treating the electric field as a qualitative feature of a band diagram, its influence should be evaluated through experimentally measurable changes in surface potential and carrier distribution. KPFM combined with spatially resolved surface photovoltage measurements has demonstrated that the surface space charge region can determine the direction of photogenerated carrier transport in individual semiconductor particles. In *n*‐type BiVO_4_ and p‐type Cu_2_O, spatially distributed photogenerated carriers were correlated with the internal electric field within the surface space charge region, demonstrating that the field can promote unidirectional carrier migration between the bulk and surface [88]. The magnitude and direction of the BIEF are strongly dependent on the electronic properties and interfacial structure of the coupled materials. Fermi‐level differences, work function mismatch, surface states, defects, and crystallographic interfaces can all modify interfacial charge redistribution and consequently alter the extent of band bending. In lattice‐matched CsPbBr_3_/CsPb_2_Br_5_ polytypic nanocrystals, KPFM measurements revealed an increase in the interfacial BIEF from 43.5 to 68.7 mV, while ultrafast transient absorption measurements identified additional interfacial carrier transfer pathways. These observations demonstrate that reducing structural mismatch and strengthening atomic scale interfacial coupling can modify the interfacial electronic potential and facilitate charge transfer [89].

Direct visualization of carrier distributions further demonstrates that BIEF‐mediated transport is not necessarily uniform across a photocatalyst surface. Electron microscopy investigations of BiOBr platelets with contiguous facet junctions revealed that photoexcited electrons were spatially redistributed according to the local internal electric fields. The measured carrier distributions could be described using diffusion and drift contributions, demonstrating that both particle dimensions and field‐dependent drift distances influence charge separation efficiency. These findings indicate that BIEF‐driven carrier transport should be considered a spatially dependent process rather than simply a binary bulk‐to‐interface migration [90].

The BIEF can also originate from ferroelectric polarization**,** where spontaneous polarization produces an internal electrostatic potential capable of directing photogenerated carriers. Spatially resolved surface photovoltage measurements of single‐domain PbTiO_3_ nanoplates demonstrated that the depolarization field can drive photogenerated electrons and holes toward opposite polarization facets. The magnitude of this driving force depends on the potential difference generated across the polarized particle, while screening fields can partially compensate the depolarization field. These observations establish polarization‐induced electric fields as another mechanism capable of controlling spatial charge separation [91]. Ferroelectric polarization can additionally interact with semiconductor interfaces to modify charge transfer pathways. In BaTiO_3_/CdS hybrid photocatalysts, the dipolar fields associated with ferroelectric BaTiO_3_ were reported to facilitate carrier migration from CdS toward the surface, illustrating how polarization‐induced electrostatic fields can regulate carrier transport across semiconductor/ferroelectric interfaces. However, such systems should be distinguished from conventional S‐scheme heterojunctions because polarization‐induced fields and Fermi‐level‐driven BIEFs can have different physical origins [92].

The influence of polarization and internal electric fields can be further modified through defect and compositional engineering. In Pr/Mn co‐doped BiFeO_3_, suppression of oxygen‐vacancy‐related defects and modification of the ferroelectric phase structure altered the polarization behavior and photoelectrochemical response. Such observations emphasize that defects can influence BIEF strength indirectly by modifying polarization, surface electronic states, and carrier density. However, changes in photocurrent or polarization alone do not establish a specific S‐scheme or Z‐scheme charge transfer pathway and should therefore be interpreted as supporting evidence for interfacial field regulation [93]. Internal electric fields can also be deliberately coupled with heterojunction formation to regulate carrier separation. In BiFeO_3_/ZnIn_2_S_4_, ferroelectric polarization and the interfacial electric field were combined to promote charge separation and carrier migration. This demonstrates that multiple electrostatic driving forces can coexist within a heterostructure and collectively influence carrier dynamics. Nevertheless, the presence of an internal electric field does not by itself distinguish a Z‐scheme from an S‐scheme; the direction of carrier migration and the origin of the field must be independently established [94].

Recent studies further demonstrate that the BIEF can substantially modify charge transfer pathways even when the interface does not conform to a conventional semiconductor–semiconductor heterojunction. In Cu‐Ni_3_S_4_/Mn_0__.__5_Cd_0__.__5_S, combined in situ XPS, KPFM, fs‐TAS, and DFT/TDDFT calculations were used to demonstrate an interfacial electric field that altered the expected carrier migration direction in a Schottky‐type architecture. This provides an important mechanistic distinction: The existence of an IEF is not sufficient to assign an S‐scheme mechanism because the field can also modify charge transport in Schottky, ferroelectric, and other interfacial systems [95].

### 3.3 Insights From Density Functional Theory (DFT) and Computational Modeling

A major advancement with DFT is the identification of charge redistribution at the interfaces and BIEF. The findings of DFT calculations determined that the joining interface of g‐C_3_N_4_/TiO_2_ undergoes charge redistribution. The surface of TiO_2_ acquires a negative charge, while the surface of g‐C_3_N_4_ acquires a positive charge. This change led to an electric field at the interface that was favorable to facilitate the transfer of electrons from the conduction band of TiO_2_ to the valence band of g‐C_3_N_4_ or the opposite. It is worth noting that the electrons left behind on g‐C_3_N_4_ are strongly reducing and the holes generated on TiO_2_ are strongly oxidizing. The theoretical indication of direct Z‐scheme configuration rather than ordinary type‐II pathway is provided by calculated band offsets and charge distribution [96]. DFT can further establish the Fermi‐level alignment and the direction of the interfacial electric field. Theoretical calculations of electronic structure and Fermi levels in the Ag_3_VO_4_/BiVO_4_ system indicated that charge redistribution occurs after contact formation due to electrical integration across the Ag_3_VO_4_/BiVO_4_ interface. The Z‐scheme pathway was subsequently supported by experimental radical trapping and EPR measurements. The combination illustrates the complementary nature of computation and experiment: DFT establishes the thermodynamic and electronic basis for interfacial charge transfer, whereas experiment is needed to confirm the photoinduced carrier pathway in practice [97].

It can also be studied in a computational way as an interfacial charge transfer bridge. In a combined theoretical and experimental study, DFT calculations and reactive species analysis were used in the Ag/AgVO_3_/carbon‐rich g‐C_3_N_4_ system to unveil why the Ag component plays the role of an electron transfer bridge in the proposed Z‐scheme architecture. Such modeling can enhance the understanding of electronic coupling between neighboring phases and determine whether an interfacial element is a plausible pathway for selective carrier recombination. Nonetheless, calculated charge distribution should not direct to a Z‐scheme mechanism a priori in the absence of experimental evidence depicting the direction of photoinduced charge migration [98]. Computational modeling could also resolve another essential parameter; the interfacial bonding. An experimental and theoretical investigation into the interfacial S─O bond formation in MnIn_2_S_4_/WO_3_ heterojunction was done. It is proposed that the interfacial charge transfer resistance is reduced due to the presence of covalent interaction, which serves the Z‐scheme carrier transfer pathway. This indicates that DFT can go beyond classical band diagrams and reveal the chemical interactions that alter the electronic coupling at the junction. Still, the presence of an interfacial bond should be regarded as a structural factor negatively impacting charge transfer rather than definitive evidence for a Z‐scheme mechanism by itself [99]. However, static DFT calculations at the SQC level are never meant to yield time evolution—only equilibrium description. The aforementioned limitation has led to an integration of DFT with nonadiabatic molecular dynamics (NAMD), which considers excited‐state carrier transfer and electron–phonon interactions. Using DFT plus NAMD at room temperature identified interfacial charge redistribution and the buildup of a BIEF at a ScP/GeS heterointerface, while time‐resolved simulations revealed an ultrafast interlayer recombination pathway consistent with a dynamically dominant Z‐scheme mechanism. These calculations offer improved mechanistic evidence relative to static band alignment as they address kinetics of photoinduced carrier evolution directly [100].

An identical computational strategy has been employed for 2D direct Z‐scheme systems. Time‐dependent ab initio NAMD simulations on a BCN/In_2_OS vdW heterostructure predicted a large interfacial electric field and ultrafast charge recombination across the interface, accompanied by competitive individual carrier transfer on a much longer timescale. Consequently, the calculations yield a kinetic basis for differentiating direct Z‐scheme recombination from interlayer type‐II carrier transfer. Notably, this approach indicates that the relative timescales of competing charge transfer pathways can provide a more rigorous mechanistic criterion than static band alignment alone [101]. The assessment of interfacial electronic properties in relation to system structural parameters using computational modeling is useful. DFT calculations in vdW heterostructures SnS_2_/WTe_2_ were performed to establish the stacking configuration, the electronic structure, band alignment, optical response, and the photocatalytic energetics. Calculations of this nature can resolve whether the band‐edge positions are thermodynamically compatible with the water splitting reactions and whether a proposed direct Z‐scheme is energetically feasible. However, band alignment and reaction energetics that are predicted should be distinguished from the experimentally demonstrated charge transfer dynamics, especially when such mechanism is implied from theory only [102]. The energetic feasibility of Z‐scheme charge transfer occurring in 2D vdW systems can similarly be evaluated by first‐principles calculations. The DFT calculations predicted low lattice mismatch and suitable electronic structure in the InN/BTe heterojunction. The Z‐scheme carrier transfer pathway electrons (reduction peak) and holes (oxidation peak) remain on the components with stronger reduction and oxidation potential, respectively. The findings demonstrate the ability of computational screening to pinpoint potential heterostructures. Theoretical prediction of a Z‐scheme pathway should thus be considered a mechanistic hypothesis in the absence of explicit experimental evidence such as interfacial charge redistribution or photoinduced carrier migration [103]. DFT and advanced computational methods lead to a multiscale understanding of charge transfer at heterojunctions, from equilibrium electronic structure and interfacial charge redistribution through to excited‐state charge carrier dynamics.

Static DFT helps to determine band offsets, work function difference, Fermi level alignment, charge density redistribution, and electrostatic potential. On the other hand, NAMD and associated time‐dependent methods can give insight into the kinetics and timescales of competing carrier transfer pathways [104]. Nevertheless, predictions from computations must not be used as evidence for a Z‐scheme. The more assurance is provided by experimental evidence such as KPFM, in situ XPS, EPR, SPV, transient absorption spectroscopy (TAS), and time‐resolved photoluminescence (TRPL), the more reliable mechanistic assignment is provided. As such, the DFT‐derived electronic structure combined with experimentally measured charge dynamics provides a more rigorous basis for distinguishing Z‐scheme charge transfer from type‐II or S‐scheme [105, 106].

## 4 Material Systems and Architectures

### 4.1 Metal Oxides and Sulfides

Metal oxides and sulfides are used to create ZSHJ photocatalysts because they offer tunability of band structures, stability, and redoxability. They have been widely used in the degradation of pollutants, generation of hydrogen, and selective conversion of chemicals. Ternary heterojunction of ZnO/Fe_2_O_3_/g‐C_3_N_4_ eliminated sulfonamide in real water and sulfamethazine 3.0 and 2.4 times faster than g‐C_3_N_4_ and ZnO/g‐C_3_N_4_, respectively. Amorphous Fe_2_O_3_ was used to mediate redox reactions between Fe(III)/Fe(II) that inhibited recombination and generated ROS (O_2_^−^, ·OH) [106]. The heterojunctions between TiO_2_ and CdS were optimized at equal weight ratio, and the yield was more than 10 times that of TiO_2_ by Z‐scheme transfer; in thin‐film form, the yield was sixfold more [107]. Based on Z‐scheme separation of charges and high conductivity, ZnO/GO composites with UiO‐66 eliminated 81% of tetracycline and 100% malathion in 90 min [108].

This system, which employed a direct Cs_2_O–Bi_2_O–ZnO Z‐scheme system, was capable of splitting water in general at 0.92% apparent quantum efficiency (AQE) at 420 nm; this was confirmed by XPS and electron spin resonance (ESR) [109]. Besides remediation, ZnAlFe‐MMO double Z‐scheme specifically converted 98% of PLA into lactic acid after 4 h incubation with ZnAlFe‐MMO catalysis under the influence of reduction of the energy barrier by Fe_2_O_3_ [110]. A dual Z‐scheme, hollow defect band‐based Bi_2_S_3_/S‐C_3_ N_3_/Bi_2_O_3_ coupled benzylamine with a yield of 97.6% and 99.4% in solar light that is driven by hollow defect bands and high‐performance charge transfer [111]. CdIn_2_S_4_/ZnS nanocomposites delivered H_2_ evolution of 3743 µmol g^−1 ^h^−1^ (59.1‐ and 14.4‐fold enhancements over pristine CdIn_2_S_4_ and ZnS) using a potent interfacial contact and Z‐scheme separation [112]. In_2_S_3_/NU‐1000 provided selective oxidation of sulfides and a simulant detoxification of sulfur mustard, the major active species being (^1^O_2_) [113]. The SnS_2_/ZnIn_2_S_4_/kaolinite heterostructure dissolved tetracycline 20.81 times faster than SnS_2_ and 2.37 times faster than ZnIn_2_S_4_ due to the increased adsorption, charge segregation, and light collection [114]. Ag^+^‐based ZnIn_2_S_4_/Bi_2_S_3_ attained 89.7% tetracycline degradation (1.87 times higher than that of pristine ZnIn_2_S_4_), and ESR was utilized to confirm the transfer of Z‐scheme using O_2_^−^ and ·OH radicals [115]. Collectively, the metal oxide/sulfide Z‐systems are highly versatile and can remove contaminants, generate solar H_2_, and valorize plastics with high activity and selectivity, as shown in Table 3.

**TABLE 3:** Representative metal oxide and sulfide‐based ZSHJ photocatalysts and their applications.

Photocatalyst system	Synthesis/design strategy	Application and performance	Reference
ZnO/Fe_2_O_3_/g‐C_3_N_4_	ZnO grafted on amorphous Fe_2_O_3_ matrix, coupled with g‐C_3_N_4_	Sulfamethazine degradation: 3.0× versus g‐C_3_N_4_, 2.4× versus ZnO/g‐C_3_N_4_; complete sulfonamide degradation in real water within 8 h	[106]
TiO_2_–CdS	Optimized TiO_2_/CdS ratio, thin‐film design	>10× H_2_ yield versus TiO_2_; thin‐film mode: 6× higher H_2_ production; Z‐scheme confirmed by radical trapping	[107]
UiO‐66@ZnO/GO, UiO‐66@TiO_2_/GO	MOF–metal oxide/graphene oxide heterojunction	Tetracycline (81%) and malathion (100%) degraded in 90 min; efficient charge transfer and broadened absorption	[108]
Cs_2_O–Bi_2_O_3_–ZnO	Solution combustion method	Overall water splitting; AQE = 0.92% @ 420 nm, no cocatalyst/sacrificial agents	[109]
ZnAlFe‐MMO (flower‐ball morphology)	Ternary oxide double Z‐scheme	98% PLA converted to lactic acid with ~100% selectivity in 4 h visible light	[110]
Bi_2_S_3_/S–C_3_N_4_/Bi_2_O_3_	Sequential adsorption, pyrolysis, sulfidation	Benzylamine conversion 97.6%, imine selectivity 99.4% in 4 h solar irradiation; hollow defect band structure	[111]
CdIn_2_S_4_/ZnS	One‐step hydrothermal method	H_2_ evolution rate: 3743 μmol g^−1 ^h^−1^ (59.1× CdIn_2_S_4_; 14.4× ZnS); confirmed Z‐scheme by VB‐XPS, Mott–Schottky, EPR	[112]
In_2_S_3_/NU‐1000	Hybrid with MOF framework	Selective sulfide → sulfoxide oxidation under full‐spectrum LED at RT; detoxification of sulfur mustard simulants	[113]
SnS_2_@ZnIn_2_S_4_@kaolinite (SZK)	Layered heterostructure with kaolinite	TC degradation rate *k* = 0.0231 min^−1^ (20.8× SnS_2_; 2.37× ZnIn_2_S_4_); pathways by HPLC–MS	[114]
Ag^+^‐doped ZnIn_2_S_4_/Bi_2_S_3_	Composite nanospheres with Ag^+^ doping	89.7% TC degradation (1.87× ZnIn_2_S_4_); Z‐scheme via O_2_^−^ and •OH radicals	[115]

### 4.2 Carbon‐Based Semiconductors (g‐C_3_N_4_, Graphene, Carbon Nanotubes, Reduced Graphene Oxide)

Graphene, rGO, CNTs, and g‐C_3_N_4_ are carbon‐based semiconductors, and they are commonly used as Z‐scheme photocatalysts since they have tunable band structures, large surface area, and high electron mobility, enabling them to separate and transfer charges to degrade pollutants and generate hydrogen. Microwave‐irradiated 2D RGO@g‐C_3_N_4_/BiVO_4_ composite was found to be dual purpose in terms of H_2_ production and degradation of tetracycline. Genesis H_2_ was produced in amounts of 6600 µmol g^−1 ^h^−1^, 10 and five times that of g‐C_3_N_4_ (660 µmol g^−1 ^h^−1^) and BiVO_4_ (1220 µmol g^−1 ^h^−1^): Photocatalyst degraded 92% tetracycline in 50 min at *k* = 0. 0982 min^−1^, about 15 times quicker than pure g‐C_3_N_4_ and BiVO_4_, as a result of the simultaneous charge transfer of BiVO_4_, rGO, and g‐C_3_N_4_ [116]. A ternary AgI/CuBi_2_O_4_/AgBr Z‐scheme showed 99.47% RhB degradation in 60 min in the presence of visible light, 120 times higher than discrete components, and 83.9% activity remained after three cycles. Electron transfer at AgBr/CuBi_2_O_4_ to AgI CB was measured using a mechanistic experimental system, with an excitation‐driven reaction yielding most importantly ·O_2_^−^ but inhibiting photocorrosion [117]. Pristine g‐C_3_N_4_ has a poor conductivity as well as a fast recombination; strategies to overcome this include doping, heterostructure design, and surface modification. CdZnS/g‐C_3_N_4_ and ZnIn_2_ S_4_/g‐C_3_N_4_ exhibited 2.75 and 1.9 times more RhB degradation than g‐C_3_N_4_, respectively. The addition of Co_3_O_4_ increased photocurrent, Ag doping the separation of the e^−^ and h^+^, Ag Cl/g C_3_N_4_ the activity of the visible light, and carbon quantum dots (CQDs) the charge transfer, reducing bandgaps and improving the pollutant removal [118].

In addition to remediation, Z‐scheme photocatalysts that are based on carbon aid in storing energy. P‐g‐C_3_N_4_/Fe_3_O_4_ nanocomposite exhibited a high Fe–C interaction, superparamagnetism, and a capacitance of 3470 mF cm^−2^ at 3 mA cm^−2^ with 74.1% remaining after 1000 cycles [119]. g‐C_3_N_4_/Co_3_O_4_ carbon‐modified biomass and magnetic recovery were used in combination [120]. CQDs in g‐C_3_N_4_/BiVO_4_ reached 85.7% benzyl paraben removal at visible light by acting through the contribution of •O_2_^−^ and ·OH [121], whereas NCQDs/BiVO_4_ enhanced RhB degradation by promoting charge separation [122]. In the case of solar‐to‐fuel conversion, a g‐CN/Ag/Ag_3_PO_4_‐AgPd photocatalyst exhibited a turnover frequency of 2107 µmol g^−1 ^h^−1^ dehydrogenation of formic acid under LED light at 50 °C [123]. An improved pCN/WFB/BiVO_4_ composite was able to reduce Cr (VI) and degrade RhB at the same time, which was catalyzed by the electron transport through biochar and an effective separation of charges [124].

### 4.3 Bismuth‐Based Compounds and Oxyhalides

Bismuth‐based semiconductors and oxyhalides are promising Z‐schemeless photocatalysts because they absorb visible light, are chemically stable, and have an appropriate band structure to achieve the separation of charges to be used in the environment and to generate energy. The synthesis of MoS_2_/Bi_2_WO_6_ (MB) heterostructures by in situ growth revealed morphology‐dependent activity; MB‐9 was able to degrade RhB (90 min) and inactivate *P.* *aeruginosa* (60 min), with h^+^ and O_2_^−^ as the most prevalent species [125]. Atrazine was degraded by CdS/BiOBr/Bi_2_O_2_CO_3_ combusted by hydrothermal means at a rate of over 95% in 30 min through a two‐step Z‐system mechanism and was less cytotoxic [21]. The 2D/2D Bi_3_TiNbO_9_/BiOBr heterojunction had a *k* = 0.0228 min^−1^ that was 6.5 and 1.5 times higher than Bi_3_TiNbO_9_ and BiOBr, respectively, with the help of DFT and active species analysis [126]. Thioacetamide‐optimized electrospun Bi_2_S_3_/BiFeO_3_ nanofibers were more active than either of the components and followed a direct Z‐scheme mechanism [127].

IO_3_^−^/I^−^ as a charge channel was used in 3D BiOIO_3_/Bi_5_O_7_I microspheres, with 98.9% removal of BPA in 60 min; degradation pathways were explained by DFT and liquid chromatography–mass spectrometry (LC–MS) [128]. AgI/Bi_24_ O_31_Cl_10_ reduced 85.36% Cr(VI) and degraded 78.26% tetracycline in 1 h with recyclability, and the Z‐scheme process was confirmed by ESR [129]. The flux method resulted in Bi_4_NbO_8_ Cl oxyhalide, which had plate‐like morphology and was three times more active in O_2_ evolution and when combined with Ru/SrTiO_3_: Rh and Fe^3+^/Fe^2+^ redox mediator allowed overall water splitting [130]. The heterostructures between BiOM_2_O_3_ and 2D/2D Bi_2_O_2_CO_3_/Bi_4_O_9_Br_2_ nanosheets demonstrated that I‐doping increased Type‐I to Z‐scheme systems and raised the capacity of the system to remove NO_8_ [131, 132]. Bi_5_O_7_I/UiO‐66‐NH_2_ heterojunction eliminated 96.1% ciprofloxacin in 120 min under white light; redox potential and environmental safety were preserved using ESR and DFT [133].

### 4.4 Perovskites and Emerging 2D/van der Waals Heterostructures

Heterostructures based on perovskite and 2D vdW materials have long been considered versatile platforms for Z‐scheme photocatalysis due to their atomic thickness and tunable electronic structures, as well as the high carrier mobility and broad light absorption. In addition, chemical modification of interfaces allows control over interfacial charge transfer processes. The use of layer‐structured materials has become increasingly important for effective charge transport and photocatalytic conversion due to the promises presented by, for example, accelerated surface phenomena through the otherwise small interfacial area in combination with Stringent control over electrical and optical properties and numerous opportunities regarding defect/chemical functionalization. These features are important for Z‐scheme architectures. In Z‐scheme architectures, interfacial electronic coupling and band alignment are required to provide selective charge recombination and simultaneously retain the strongly reducing and oxidizing carriers required for water splitting, CO_2_ reduction, and pollutant degradation. However, the actual use of 2D types of photocatalysts is restricted due to factors involving interfacial stability, scalable synthesis, reproducibility, and efficient charge transport during operation [134]. The stability, direct bandgaps, high carrier mobility, and strong visible–UV absorption of BAs/Janus MXY (M = W; X/Y = Se, Te) heterostructures and BAs/WTeSe (with Se vacancies) in particular were very encouraging. Ti_2_CO_2_‐WSe_2_ bilayers exhibited long carrier lives (1.13–1.22 ps), allowing efficient Z‐scheme transfer as well as spontaneous HER/OER in the absence of cocatalysts [135]. LiInP_2_S_6_/XTe_2_ (X = Mo, W) vdW systems were shown to exhibit indirect‐to‐direct band transitions, visible absorption, and mobilities as high as 4.21 × 10^3^ cm^2^·V^−1^·s^−1^ and interfacial fields that favored water redox reactions [136]. MX_2_/MSSe (M = Ge, Sn; X = S, Se) heterostructures had intrinsic electric fields, tunable bandgaps, broad absorption, and mobilities of 10^4^ cm^2^·V^−1^·s^−1^; in particular, T‐phase Sn‐based structures preferred spontaneous water splitting [137]. Besides, g‐ZnO/SnXY (X, Y = S, Se) strain and electric field engineered attained high solar‐to‐hydrogen efficiency at different pH, and near‐free‐electron states improved carrier transport [138].

Z‐schemes also make use of perovskite heterostructures. It was demonstrated that LaFeO_3_/g‐C_3_N_4_ p–n systems exhibited 16.9‐ and 7.8‐fold improved Brilliant Blue degradation relative to the untouched g‐C_3_N_4_ and LaFeO_3,_ respectively [139]. LaFeO_3_/In_2_S_3_ heterojunctions were able to realize degradation rate constants of 0.16709 min^−1^ (RhB), 0.02684 min^−1^ (TCH), and 0.0175 min^−1^ (BB) through the ROS trapping and work function modulation [140]. Narrowed bandgaps, an increased surface area, and effective dye degradation by the h^+^, ·O_2_^−^, and ·OH pathways were observed in SrTiO_3_/g‐C_3_N_4_‐Ag composites as a result of scavenger tests [141]. Bi co‐sharing resulted in the production of lead‐free Cs_3_Bi_2_I_9_/Bi_2_WO_6_ heterojunctions, exhibiting direct Z‐scheme transfer and fourfold improvement of CO_2_ to CO conversion rate over Cs_3_Bi_2_I_9_, combining rational design with high photocatalytic rate [142].

Figure 3 indicates the representatives of perovskite and 2D/vdW Z‐schemes: (a) atom and band structures of 2D SnC and SnS, indicating their semiconducting properties and the potential to serve as components of a vdW Z‐schemes [143], (b) vdW heterojunctions using Ga_2_Se monolayers with GaP, PtSSe, and SnSSe monolayers under one of the arrangements, which showcase enhanced pollutant degradation under sunlight [144], (c) band mapping of GaN/g‐C_3_N_4_, revealing position of band edges that can be used to decompose water by Z‐scheme charge transfer [145], (d) type‐II versus direct Z‐scheme charge transfer processes in NiMoO_4_ /ZnFe_2_O_4_, which demonstrated an improved rate of pollutant degradation by sunlight [146], and (e) MAPbI_3_ and Z‐scheme MAPbI_3_/Fe_2_O_3_ structural configurations and molecular dynamics simulations, which showed better interfacial stability and charge transfer for photocatalysis [147].

**FIGURE 3:**
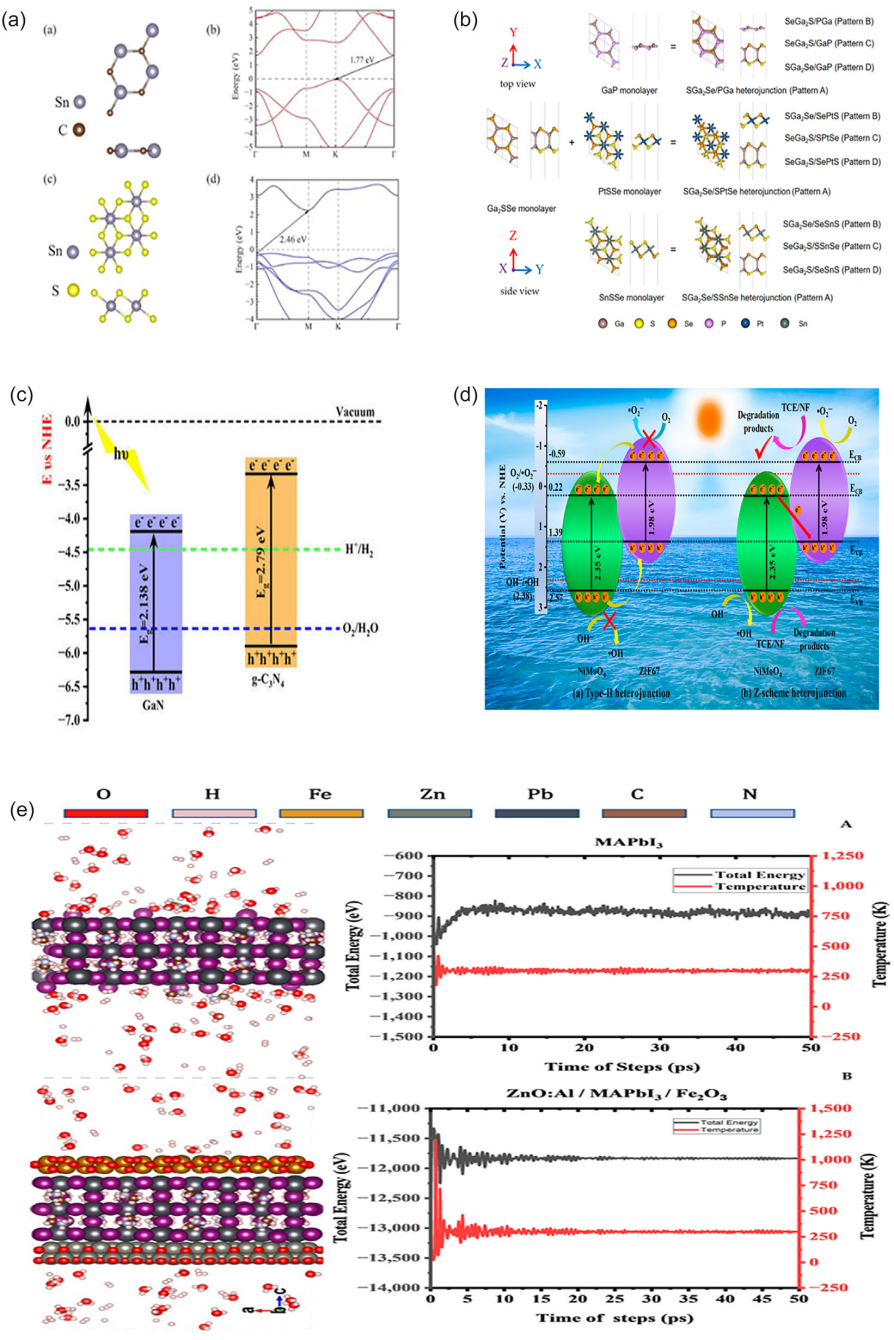
(a) Atomic structures and band structure of 2D SnC (a,b) and SnS (c,d), which shows the characteristics of a semiconductor and its possible usage as a vdW heterostructure component, Z‐scheme [143]. (b) Ga_2_Se monolayer‐based vdW heterojunctions with monolayers of GaP, PtSSe, and SnSSe with various stacking patterns and configurations as potential Z‐scheme potential Z‐scheme photocatalytic applications [144]. (c) Band alignment of GaN and g‐C_3_N_4_ showing band edge positions and possible position of a ZSHJ to split water photocatalytically [145]. (d) Type‐II and direct ZSHJ mechanisms in NiMoO_4_/ZnFe_2_O_4_, using comparison to illustrate the charge transfer pathway and increased degradation in pollutants when under a sun under sunlight [146]. (e) Structural configurations and molecular dynamics simulations of MAPbI_3_ and Z‐scheme MAPbI_3_/Fe_2_O_3_ heterojunctions, which exhibit enhanced interfacial stability and charge transfer to photocatalytic use [147].

### 4.5 Ternary and Quaternary Z‐Scheme Systems

The secondary tasks of photocatalysis are high photoelectric conversion efficiency and great redox ability that have not been reached yet. The solution to this lies in ternary and quaternary ZSHJs that allow the rapid charge separation, long carrier lifetimes, and a wide range of visible‐light absorption. An UV‐irradiated O‐g‐C_3_N_4_/Zn_2_SnO_4_N/ZnO heterojunction demonstrated enhanced dye and NO degradation as a result of two in‐built electric fields and interfacial bonds [148]. A ternary heterostructure of CdS/BiOBr/Bi_2_O_2_CO_3_ synthesized by hydrothermal method demonstrated atrazine degradation in excess of 95% within 30 min, and a two‐step Z‐scheme mechanism as reported by photoelectrochemical and radical trapping studies [21]. Similarly, g‐C_3_N_4_ at Bi/BiOBr hierarchical composites [149], Ag_3_PO_4_/AgBr/g‐C_3_N_4_ dual Z‐scheme systems [150], and CN/MS/BOC (g‐C_3_N_4_ /MoS_2_/Bi_24_O_31_Cl_10_) heterojunctions [151] had improved carrier separation and photocatalytic degradation of antibiotics and dyes via indirect or dual Z‐scheme transfer.

These benefits are extended by quaternary systems. The RhB removal efficiencies of CuO–CuS–ZnO–ZnS nanocomposites were 34 and 2.9 times greater than pristine CuO and ZnS, with 99.17% sulfamethoxazole removal at a *k* value of 0.0380 min^−1^ occurring through the use of two‐step Z‐scheme transfer [152] and 99.17% removal at 0.0380 min^−1^ in CuO/Cu_2_O/Cu [153]. The ZnO @ZnS/Ag_2_S system had high H_2_ evolution (140.3 µmol g^−1^ in 5 h), which is higher than the binary and ternary systems [154]. The BiOBr/Bi_2_O_3_ bare or Ag/AgBr‐decorated photocatalysts were found to provide almost absolute degradation of tetracycline, methyl orange, and rhodamine B due to synergistic plasmonic as well as Z‐scheme effects [155]. Polymorphic chalcogenides of ZnIn_2_S_4_ further underscore the significance of structural control in improving Z‐scheme photocatalysis, all hexagonal polymorphs of which have a preferred band alignment to water redox reactions [156].

## 5 Synthesis and Interface Engineering Strategies

### 5.1 In Situ Growth and Heteroepitaxy

MOF‐based heterojunctions can provide an alternative pathway for regulating interfacial charge transport as their porous architectures, tunable coordination environments, and chemically adjustable linkers allow the simultaneous control of surface adsorption, electronic structure, and interfacial contact. The recent advances in porous materials show that rational control of pore architecture, framework composition, defect chemistry, and interfacial connectivity are required to transform high surface area into efficient charge separation and photocatalytic conversion. In Z‐scheme architectures, these structural parameters should accordingly be assessed in conjunction with band alignment and interfacial electronic coupling rather than regarded as simply factors that enhance surface area. The integration is needed for CO_2_ conversion, water splitting, H_2_O_2_ generation, pollutant degradation, etc., where mass transport and interfacial carrier migration operate simultaneously [157]. The 94.8% and p‐nitrophenol removal rates of the 2‐g heterojunction 2–3 and 4‐B‐Bi_2_O_3_ were found to be 7.1 times higher than that of the individual components, confirming Z‐scheme transfer [158]. Similarly, V_2_O_5_/g‐C_3_N_4_ systems were found to be 713 times more active with several dyes than parent systems and ESR and trapping measurements verify that these systems retain effective separation of carriers [159].

Heterojunctions of hydrothermal CuInS_2_/Bi_2_WO_6_ exhibited an enhanced TCH degradation in pharmaceutical wastewater with a greater than threefold improvement and 90.5% chemical oxygen demand (COD) removal with good stability [160]. SnS_2_/BiOBr too showed rapid charge transfer with h^+^ and ·O_2_^−^ dominating in RhB degradation [161]. Large solar‐to‐chemical conversion has also been made possible by heteroepitaxy. ZnO/CdS heterostructures were regrown by two‐step epitaxy, and it gave 669.6 μmol g^−1 ^h^−1^ H_2_ [100 mg] without any cocatalysts, and also, it was very stable [162]. MOF‐on‐MOFs, such as MIL‐88B(Fe)/UiO‐66‐NH_2_, were claimed to achieve nearly 100% CO‐to‐CO selectivity [163] and MIL‐88B(Fe)/UiO‐66(Zr)‐NH_2_ nearly 0.9% overall water splitting [164]. Compared to ZnO and ZnO–Au, ZnO–Au@CdS nanorod arrays prepared by a vectorial Z‐scheme design were 79‐ and 28‐fold higher activity in selective nitro reduction and had a high recyclability [165]. Likewise, the Au nanorod‐covered SnO_2_/TiO_2_ nanorods improved two‐electron O_2_ reduction through LSPR‐mediated vectorial delivery (Au–TiO_2_–SnO_2_–Au) [166]. The combination of these results confirms that in situ growth and heteroepitaxial approaches provide strong interfaces and effective charge transportation, as shown in Table 4, which allow conversion of sustainable energy and remediation of the environment.

**TABLE 4:** Representative in situ growth and heteroepitaxial ZSHJ photocatalysts and their applications.

Photocatalyst system	Fabrication strategy	Application and performance	Reference
Bi_7_O_9_I_3_/g‐C_3_N_4_	In situ growth on ultrathin g‐C_3_N_4_	Doxycycline degradation; oxhydryl and O_2_^−^ as main species; stable and recyclable	[157]
β‐Bi_2_O_3_/g‐C_3_N_4_ (0.5BC)	In situ growth; nanosheets coating g‐C_3_N_4_	RhB (94.8%/120 min) and p‐NP (71.6%/300 min); *k* = 5.8–7.1× higher than single components	[158]
V_2_O_5_/g‐C_3_N_4_ (VC1.0%)	In situ solid‐state growth	Degradation of RhB, TC, MO, MB; efficiencies 7.3× versus g‐C_3_N_4_, 13× versus V_2_O_5_	[159]
CuInS_2_/Bi_2_WO_6_	In situ hydrothermal growth	TC degradation >3× CuInS_2_; COD removal 90.5% in real wastewater; excellent stability	[160]
SnS_2_/BiOBr	In situ growth of BiOBr nanosheets on SnS_2_	RhB degradation; h^+^ and O_2_^−^ as dominant active species; improved charge transfer	[161]
ZnO/CdS	Two‐step heteroepitaxial growth (anion exchange)	H_2_ production 669.6 μmol h^−1^ (100 mg); cocatalyst‐free; stable 72 h	[162]
MIL‐88B(Fe)/UiO‐66‐NH_2_	MOF‐on‐MOF heteroepitaxial lattice growth	CO_2 _→ CO photoreduction (100% selectivity; 2.26 μmol g^−1 ^h^−1^)	[163]
MIL‐88B(Fe)/UiO‐66(Zr)–NH_2_	Face‐selective epitaxial growth	Overall water splitting; AQY ≈ 0.9% (400–450 nm)	[164]
ZnO–Au@CdS nanorod arrays	Heteroepitaxial core–shell nanorod growth	Selective reduction of nitro compounds; 79× ZnO, 28× ZnO–Au; stable and recyclable	[165]
Au/[SnO_2_–NR#TiO_2_]	Heteroepitaxial SnO_2_/TiO_2_ nanorods + Au NPs	Two‐electron O_2_ reduction; vectorial electron transfer via Au–TiO_2_–SnO_2_–Au; high efficiency	[166]

### 5.2 Atomic Layer Deposition and Thin‐Film Engineering

By the atomic layer deposition (ALD) and thin‐film engineering, interfaces, thickness, and composition can be controlled and thin‐film heterojunctions can be engineered to be Z‐schemed with enhanced charge separation and light absorption and recoverability. ALD synthesis of a TiO_2−*x*_/Cu/ZnO heterojunction and spray pyrolysis (without noble metal cocatalysts) showed a high level of SPR absorption and high H_2_ evolution (342.0 µmol g^−1 ^h^−1^) [167]. ZnS QDs deposited using ALD on g‐C_3_N_4_ increased the activity 2.6‐fold, with the best deposition cycles reducing recombination [168]. On the same note, 3D ZnO on 2D g‐C_3_N_4_ demonstrated 98.9% cephalexin degradation with h^+^ and ·OH as the prevailing species [169].

Heterojunctions of Ta_3_N_5_WO_2.72_ prepared through ALD sol–gel increased the H_2_ yield by up to 10‐fold relative to Ta_3_N_5_ and increased it by >10‐fold relative to Z‐schemes in the liquid state [170]. Oxygen vacancies and internal electric fields facilitated the degradation of levofloxacin 89% within 30 min with the assistance of the SiO_2_/TiO_2_/Bi_2_WO_6_ heterojunction [171]. g‐C_3_N_4_/TiO_2_ multilayers immobilized on glass were found to have high stability and efficiency in RhB and TCH degradation [59].

Thin films that were prepared using SILAR CdS/BiOI/WO_3_ had 98.3% MB degradation with a 118.4 times faster kinetics compared to WO_3_ [172]. The bilayers of TiO_2_/BiVO_4_ with Bi nanoparticles used modified SPR and Z‐scheme transfer in RhB degradation [173]. Photoanodes made of CN‐BiVO_4_/WO_3_ showed 14.8% incident photon‐to‐current efficiency (IPCE) at 390 nm and improved stability [174], and FeO*_x_*/g‐C_3_N_4_ thin films (PLD‐grown) were strong in terms of photo‐Fenton and photocatalytic ciprofloxacin degradation [175]. All in all, ALD and thin‐film engineering provide accurate structural control and interfacial optimization, which allow efficient, stable, and recyclable Z‐scheme photocatalysts to be used in energy and environmental use cases.

### 5.3 Defect/Vacancy Customization and Surface Functionalization

Defect engineering and surface functionalization can greatly overcome the drawbacks of ZSHJs, improving light absorption, charge separation, and surface reaction kinetics, especially in 2D materials with large surface area and high density of active sites. In situ grown ultrathin ZnIn_2_S_4_/g‐C_3_N_4_ heterojunctions demonstrated effective H_2_ evolution in the absence of Pt with DFT and spectroscopy, showing that charge separation was the crucial parameter [81]. G‐C_3_N_4_/C‐TiO_2_ composites reported oxygen‐vacancy‐assisted transfer with six‐ to sevenfold higher RhB and phenol degradation compared to TiO_2_ [176]. Surface‐modified g‐C_3_N_4_/ZnSe was better at adsorbing and transferring CO_2_, with selectivity of 88% to CO (439 µmol g^−1^) and CH_4_ (203 µmol g^−1^) [177].

Binary Bi_2_WO_6_/TiO_2_ was able to produce H_2_ at 12.9 mmol/g^−1 ^h^−1^, 129‐fold higher than TiO_2_ [178], and Bi_2_ MoO_6_/BIOBr demonstrated 4–7 times greater MB degradation rate (*K*_ap__p_ = 0.0539 min^−1^) [179]. AgI/Ce‐2MI nanocomposites were able to reduce over 90% Cr(VI) in the view of the visible light, and •O_2_^−^ was confirmed to be produced through ESR [180]. B‐BiVO_4_/Bi_2_MO_6_‐β systems with recombination suppressed were obtained by co‐doping and by two‐phase modulation [181]. The incorporation of cocatalysts enhanced the performance even more: Ternary TiO_2_/g‐C_3_N_4_/NiCoP double Z‐schemes provided 2305.5 mmol/g^−1^ H_2_ removal and 86.2 Cr(VI) removal in 2 h [182]. AgI/Bi_2_WO_6_ (20 wt% AgI) exhibited a 5.4‐ and 3.3‐fold higher tetracycline degradation than Bi_2_WO_6_ and AgI, respectively, which is facilitated through the formation of in situ Ag nanoparticles [183]. Hence, carrier dynamics, expansion of light harvesting, and surface reactivity are effectively controlled using defect/vacancy tailoring and surface functionalization to improve Z‐scheme photocatalysts in solar fuel generation and environmental cleaning.

### 5.4 Noble Versus Earth‐Abundant Mediators

Noble metals (Pt, Au, Pd) are good HER cocatalysts but are costly and scarce, and there is research on earth‐abundant alternatives. An efficient H_2_ evolution of a ternary Bi/Bi_5_O_7_I/Sn_3_O_4_ photocatalyst which does not contain noble metals, via direct Z‐scheme charge transfer, was enabled by Bi, which extended the absorption and acted as a cocatalyst [184]. Fe_3_O_4_@NiFe_2_O_4_/P‐doped g‐C_3_N_4_ (M @NFOPCN) produced 924 µmol h^−1^ of H_2_ and twofold Z‐scheme separation Z‐schemes and electric field effects doubled the degradation of ciprofloxacin [185]. S‐deficient CoS/CdS nanoplates generated 39.29 mmol·g^−1 ^h^−1^ of H_2_ with 14.5% AQY at 400 nm, which was superior to 3 wt% Pt ‐CdS [186].

Composites of TiO_2_–Cu@C employed carbon dots as the mediator and yielded 3911 µmol g^−1 ^h^−1^ of H_2_ [187] and CuInS_2_/CN*x*–CN*x* with Au plasmonic enhancement with 10.72 mmol g^−1 ^h^−1^ of H_2_ [188]. At 500 nm, Cu 3P‐CNTs were 17.22 mmol g^−1 ^h^−1^ with 10.23% AQE [189], and BP/MoS_2_ and BP/WS_2_ heterojunctions had high solar‐ and NIR‐driven H_2_ generation [190]. Z‐schemes of engineered black TiO_2_ and TiO_2_/‐C_3_N_4_ were improved in visible‐light quantum efficiency [191]. Pt‐decorated TiO_2_ heterojunctions reached 45.6% AQY at 365 nm, and noble metal efficiency but low‐cost alternatives were urgently needed [191]. TMDs (MoS_2_, WSe_2_) presented conductive and stable cocatalysts; nanosheets of WSe_2_ on CdS nanorods enhanced the charge separation and H_2_ activity [192]. Metals that were abundant in the earth also promised, Sn/TiO_2_ photocatalyst generated 553.1 µmol·g^−1^·h^−1^ of H_2_, 43.9‐pure TiO, with Sn serving as an electron sink and H_2_ generation site [193]. On the whole, earth‐abundant mediators (Ni, Co, Cu, Fe, Bi, Sn, TMDs) provide competitive or even better performance in HER, which is scalable and sustainable as an alternative to noble metals in Z‐scheme photocatalysts.

### 5.5 Polymetric and Organic Linker‐Based Methods

The applications of polymeric and COFs are so vast that they can be tailored to tune photocatalytic interfaces. The molecular backbone, linkage chemistry, pore structure, π‐conjugation, and D–A configuration can be designed deliberately to modify light harvesting, charge mobility, interfacial coupling, and catalytic site accessibility. In comparison to traditional inorganic semiconductors, COFs offer control of electronic structure and porous environments at the molecular level, allowing for the photothermal response to be tuned through linker selection, functional group incorporation, framework topology, and heterojunction construction. These features are particularly pertinent to Z‐/S‐scheme architectures, where the interface must allow for selective carrier transfer at the same time as retaining the high‐energy electrons and holes required for reduction and oxidation processes. Therefore, the photocatalytic advantage of COF‐containing heterojunctions cannot be ascribed solely to their extensive surface area; instead, a correlation among framework structure, interfacial electronic coupling, carrier dynamics, and reactant transport should be established for a quantitative structure–activity relationship [194]. In situ growth strategies that directly control the contact between the two semiconductor phases further illustrate the significance of intimate interfacial architecture. For instance, ZnCo_2_O_4_ was anchored on ZnO with the help of an in situ growth process to obtain a ZnCo_2_O_4_/ZnO S‐scheme heterojunction. The composite exhibited improved visible‐light response and carrier transfer properties for 92.64% inhibition of E. Compared to pristine ZnO, the degradation of ZnO@MnO_2_‐10 is more efficient [195]. SrTiO_3_/g‐C_3_N_4_‐Ag composites had lower bandgaps and total methylene blue degradation through a Z‐scheme pathway [141]. CPDs/Bi_4_O_8_Br_2_ (8 wt%) produced 132.42 µmol g^−1 ^h^−1^ of CO, 5.43 times greater than pure Bi_4_O_8_Br_2_, through the up‐conversion of wavelengths above 580 nm [196]. A heterostructure made of g‐C_3_N_4_ and alpha‐Fe_2_O_3_ was effective in removing 90% of HPAM present in wastewater because of effective interfacial charge transfer to produce ·OH radicals [197]. Also enhanced performances are linkers‐aided quantum dot (QD) decoration. CdS‐Bi_2_S_3_/KNbO_3_ using starch/thioglycolic acid linkers increased phenol degradation [198], and CdS‐Bi_2_S_3_/NaNbO_3_ had a low onset potential [199]. Under the light of visible light, the Au–CeO_2_/PCN possesses composites with a rate of H_2_ of approximately 80 µmol h^−1^ of H_2_ [200]. On the same note, organic linkers provided faster electron transfer: CdSe QDs–TiO_2_ with para‐4MBA exhibited better charge transfer efficiency than ortho/meta analogs [201], and DMSA‐based CuS/TiO_2_ had consistent TiO_2_@CuS core/shell structures with increased photocatalytic activity than direct deposition protocols [202].

In general, polymeric and organic linker‐based methods provide control over band deployment, strong interfaces, and design flexibility in Z‐schemes to convert solar fuel and clean up the environment. Figure 4 shows some representative systems: (a) PANi/ZnO composites contrasting type‐II and Z‐scheme charge transfer [203], (b) demonstrating the ability to promote photocatalysis through polymer [204], (c) demonstrating the ability to promote contaminant degradation through heterojunction [205], and (d) demonstrating the ability to promote tetracycline hydrochloride degradation with sunlight [206].

**FIGURE 4:**
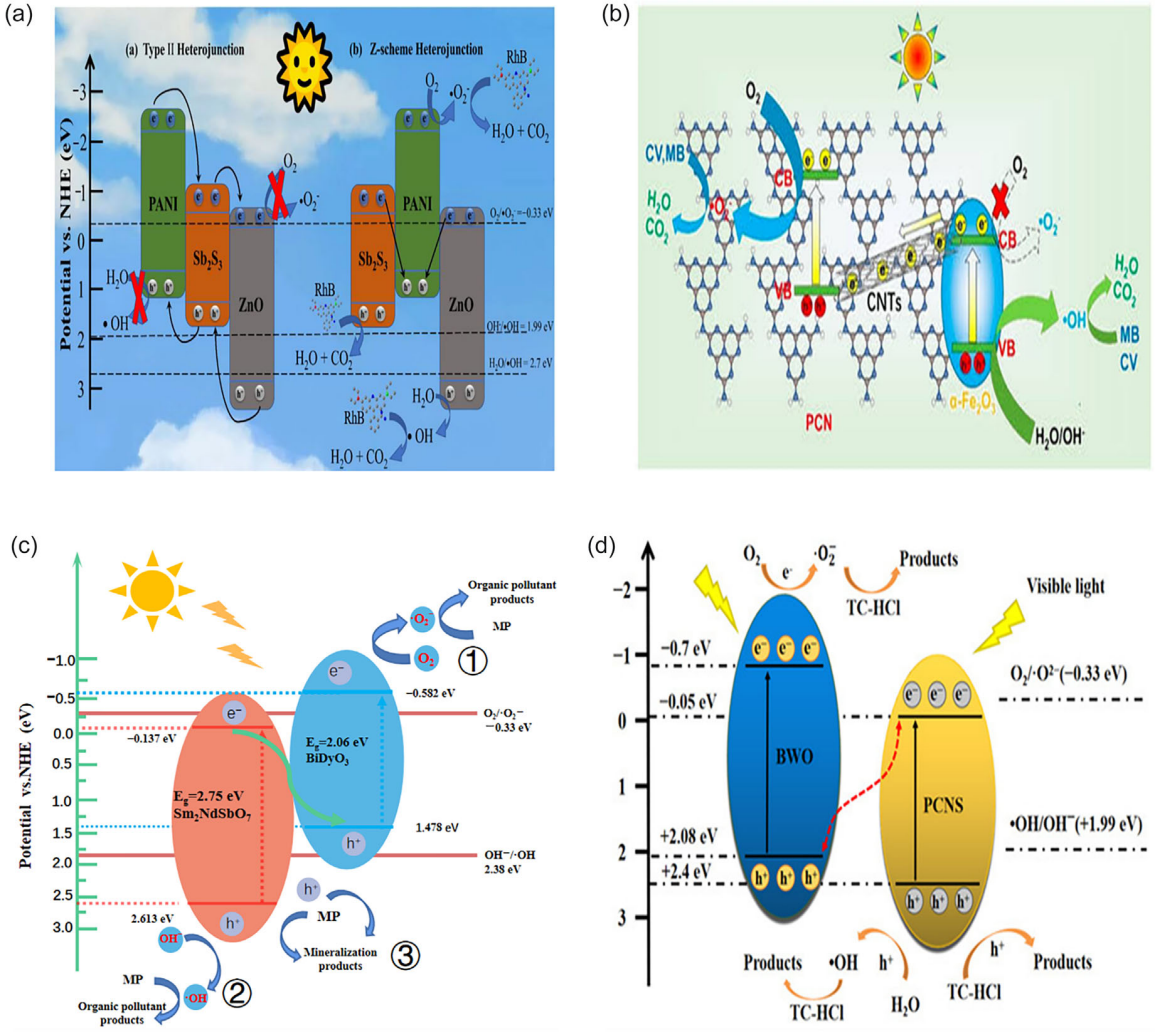
(a) Diagrammatic comparison of the type‐II and Z‐scheme charge transfer mechanisms in PANi/ZnO composites, showing improved photocatalytic behavior of polymer‐assisted heterojunction design [203]. (b) Z‐scheme charge transfers at PCN/α‐Fe_2_O_3_ heterojunction by CNTs with efficient separation of charge carriers and promoted photocatalytic degradation of organic pollutant [204]. (c) The band positioning and charge transfer mechanism of Sb_2_S_3_/Bi_2_MoO_6_ heterojunction, which shows efficient carrier movement and degradation of organic contaminants under the sun irradiation [205]. (d) Z‐charge transfer mechanism in BWO/PCNS heterojunction at the visible light, which exhibits efficient electron–holes separation and TC‐HCl photocatalytic degradation [206].

## 6 Advanced Characterization Techniques for Mechanistic Validation

### 6.1 Steady‐State and Time‐Resolved Photoluminescence (PL)

PL spectroscopy time‐resolved and steady‐state are needed to investigate the dynamics of the charge carriers in ZSHJs, and a direct correlation of the carrier lifetimes and separation efficiency to photocatalytic performance is required. To generate hydrogen using solar energy, CoTiO_3_/TiO_2_ heterostructures exhibited improved carrier lifetimes as indicated by UPS and UV–vis, which illustrated a direct Z‐scheme reaction and high hydrogen evolution capability. Performance was also further optimized by temperature modulation, which proposed the possibility of thermophotocatalytic reactors [207]. Only static band structure data cannot provide the real direction and kinetics of charge transfer. A complete mechanistic assignment must therefore include complementary characterization of the equilibrium electronic structure, interfacial charge redistribution, photoinduced carrier migration, and reactive species formation. Among others, core‐level shifts induced by illumination can be tracked by ISIXPS and thus provide direct information on changes in surface electron density, while KPFM provides spatially resolved work function and surface potential information associated with interfacial charge redistribution. By resolving the temporal evolution of excited‐state carriers, fs‐TAS complements these techniques. EPR identifies the reactive radical species produced by surviving redox‐active carriers. Recent studies of COF‐based S‐scheme photocatalysts show that ISIXPS, KPFM, fs‐TAS, as well as DFT analyses provide significantly stronger mechanistic evidence than a single band diagram or photocatalytic activity measurement [208]. Oxygen vacuum heterostructures between BCN and TiO_2_ also gave longer visible absorption and carrier lifetimes with H_2_ evolution rates 711 times longer than in TiO_2_ and BCN [209]. Composites of COF‐366‐Co/UiO‐66‐NH_2_ exhibited long lifetimes and delocalization with the generation of CO_2_ photoreduction rates of 4092 µmol g^−1 ^h^−1^ With 2.4% quantum yield at 400 nm [210]. To reduce oxygen, UiO/IKCN heterojunctions produced H_2_O_2_ at 72.6 mM g^−1 ^h^−1^ with 10.28% AQY at 420 nm. Z‐scheme charge transfer was validated using PL, transient absorption, and DFT [211]. Mediator‐free Ag_3_VO_4_/BiVO_4_ microspheres used built‐in fields, and they last longer and allow reduction of Cr^6+^ by 74.9% and oxidation of bisphenol S by 94.8% [4]

Even 0D/2D systems increased Z‐scheme transfer: Bi_3_TaO_7_ QDs/g‐C_3_N_4_ exerted ciprofloxacin degradation 4–12.2 times higher than that of individual components [212], and ZnS/ZnIn_2_S_4_ with interfaces induced by microstrains increased CO_2_ reduction efficiency [213]. Methylene blue degradation was 2.5–2.7 times more effective in composite WO_3_/g‐C_3_N_4_ systems than in pure components and was confirmed by PL to be efficiently separated [214]. Equally, g‐C_3_N_4_/Bi_4_NbO_8_Cl heterojunctions prepared by ball milling showed H_2_ evolution rates of 6.9–67.2 higher than those of single phases [215]. In general, the Z‐scheme charge transfer is established in PL studies to show the significance of heterojunction design and interface engineering, as well as mediator strategies, to achieve the maximum efficiency in the production of solar fuel and the removal of pollutants.

### 6.2 Transient Absorption Spectroscopy (Ultrafast to Microsecond Scales)

TAS is a technique that shows carrier lifetimes, carrier mobility, and recombination at ZSHJs and directly relates dynamism to photocatalytic performance. Hot‐electron transfer between g‐C_3_N_4_ excitonic states to ZIS took place in CN/ZnIn_2_S_4_ within sub‐ps, faster than that of band‐edge transfer and enhancing H_2_ evolution rate [216]. BiVO_4_V_2_O_5_ photoanodes had longer carrier lifetimes and a slower recombination rate, and the holes were trapped in BiVO_4_, proving type‐II recombination suppression [217]. In shallow traps, C_3_N_4_ crystal exposed 59.5% of the electrons to Pt in 1.6 ps (compared to 21.3% at 11.8 ps for pure C_3_N_4_), allowing 28‐fold higher evolution of H_2_ [218].

Mixed perovskite heterojunctions also proved to be advantageous: FAPbBr_3_/Bi_2_WO_6_ was able to transfer charge in ultrafast IR‐TAS, improving CO_2_ reduction 8.3‐fold over CsPbBr_3_, with simultaneous oxidation of benzyl alcohols [219, 220]. CoNiHHTP MOF/PHI in environmental sensing, Z‐scheme transfer, and bimetallic reaction contributions were demonstrated with a 1 ppb NO_2_ detection limit with fast response, TAS, and in situ measurements were consistent with CoNiHHTP MOF/PHI [221]. On the same note, WO_3_/TiO_2_ core shell heterojunctions displayed microsecond scale separations with electron transfer (WO_3_/TIO_2_) and hole transfer (TiO_2_/WO_3_), which allowed the effective degradation of stearic acid [222]. Surprisingly, In_2_O_3_‐cube/g‐C_3_N_4_ systems localized hot spots were generated, with ultrafast coupling and shallow‐trap lifetimes enhancing 34‐fold increased H_2_ production [223].

Carrier dynamics were also optimized using doping solutions: RhTiO_3_‐La and RhTiO_3_‐codoped SrTiO_3_ exhibited Rh^3+^:STO transfer in 1.6 ps and quantum yield of 14.7% [224]. In a similar way, CdIn_2_S_4_/NC/MoC heterojunctions optimized trap states, and a 58.2‐fold increase in H_2_ evolution was confirmed by fs‐TAS [225]. Taken together, TAS in the ultrafast‐to‐microsecond regime offers important information about charge generation, migration, and recombination in Z‐scheme photocatalysts, as is the case in Table 5, and that can guide the rational design of systems that work with solar fuels and pollutant remediation.

**TABLE 5:** Summary of TAS insights into charge carrier dynamics in ZSHJ photocatalysts.

System/heterojunction	TAS findings	Photocatalytic/functional outcome	Reference
CN/ZnIn_2_S_4_ (ZIS)	Sub‐ps migration of hot electrons from g‐C_3_N_4_ to ZIS; trap‐state mediated electron delocalization	Enhanced H_2_ evolution efficiency	[216]
BiVO_4_/V_2_O_5_ (Type II)	Prolonged carrier lifetimes; trapped holes mainly in BiVO_4_	Improved photocurrent density, suppressed recombination	[217]
Crystalline g‐C_3_N_4_ versus pristine	Faster electron transfer to Pt (59.5% in 1.6 ps vs. 21.3% in 11.8 ps); shallow‐trap assistance	28× increase in H_2_ evolution	[218]
FAPbBr_3_/Bi_2_WO_6_	Ultrafast Z‐scheme charge transfer confirmed	CO_2_ reduction + benzyl alcohol oxidation under solar light	[219]
α‐Fe_2_O_3_/Amine‐RGO/CsPbBr_3_	Prolonged carrier lifetime; efficient spatial separation	8.3× enhanced CO_2_ reduction efficiency	[220]
CoNiHHTP MOF/PHI	Efficient Z‐scheme charge transfer; bimetallic site synergy	NO_2_ detection limit 1 ppb (fast response/recovery)	[221]
WO_3_/TiO_2_ core–shell	μs‐scale carrier lifetimes; electron transfer WO_3 _→ TiO_2_ and hole transfer TiO_2 _→ WO_3_	High photocatalytic efficiency for stearic acid degradation	[222]
In_2_O_3_‐cube/g‐C_3_N_4_	Near‐field coupling creates “hot areas”; extended shallow‐trapped state lifetimes	34× improvement in H_2_ production	[223]
La‐ and Rh‐codoped SrTiO_3_	Rh valence controls electron lifetime; Rh^3+^:STO with 1.6 ps transfer, 14.7% QY	Efficient electron transfer to Ru cocatalyst	[224]
CdIn_2_S_4_/NC/MoC	Dynamic trap state modulation; optimized interfacial migration	58.2× higher H_2_ evolution	[225]

### 6.3 Isotopic Labeling and Reactive Species Tracking

The use of isotopic labeling and reactive species scrambling is crucial in the elucidation of charge transfer, active sites, and product origins in Z‐scheme photocatalysts. The heterostructures of BiVO_4_/Bi_2_S_3_ exhibited direct system transfer of Z‐schemes through delocalized electrons and internal fields; MnOx/BiVO_4_/Bi_2_S_3_ selective CO_2_ to methanol was possible with stoichiometric O_2_ by isotopic labeling [226]. CoAl‐LDHs with Bi_2_O_3_ made visible light fixation of N_2_ possible (48.7 µmol L^−1 ^h^−1^); N_2_ was the source, and Co was the active site, detected by EPR/DFT [227]. CO_2_ to CH_4_ (184 µmol g^−1^) and CO (105 µmol g^−1^) were also reduced by CsPbBr_3_/g‐C_3_N_4_ nanosheets, with isotopic analyses showing water‐derived H atoms [228]. Standardization of the ^15^N protocols also provided a high degree of reliability in the studies of N_2_ fixation [229].

Ternary CNC/Bi/BiOCl produced CO (8.76 µmol g^−1 ^h^−1^, approximately 100% selectivity); isotopic labeling, DRIFTS, and XPS indicated that the rate limiting step was the production of COOH [230]. Plasmonic Au‐PMTiNTs exhibited wavelength‐dependent CO_2_ reduction: CH_4_ (302 µmol g^−1 ^h^−1^, 89.3% selectivity) reduction under AM1.5G and labeling of ions with 13C verified the plasmonic Z‐schemes pathways [231]. To be degraded, in the case of the pollutants, CDs/g‐C_3_N_4_/MoO_3_ increased tetracycline degradation 46.2 times with scavenging, showing holes as the major species [232]. COF/TiO_2_ heterojunctions selectively oxidized NO (64.5% removal, 99.9% NO_3_^−^) through ·O_2_^−^ radicals and inhibited ·OH [233]. Biochar@ZnFe_2_O_4_/BiOBr and g‐C_3_N_4_/Bp/MoS_2_ systems were able to degrade antibiotics effectively, and holes and ·O_2_^−^ were proved as the dominant species, as indicated by ESR and LC–MS/MS mapping [234, 235]. LaFeO_3_/Cs catalyzed the degradation of tetracycline (88.7% in 40 min), using ·O_2_^−^, and ·SO_4_^−^, proven by ESR [236]. In general, the use of isotopic labeling and species tracking can demonstrate the origins of products, reactive intermediates, and interfacial processes, which provide mechanistic confirmation on Z‐scheme designs in solar fuels and pollutant remediation.

### 6.4 Combined Experimental–Theoretical Approaches

Rational design of ZSHJs involves semiconductors with equal Fermi levels, high absorption in the visible light, and designed interfaces. Simultaneous experimental/theoretical experiments can offer important information on the charge transfer, interfacial reactions, and the photocatalytic reactions. Theoretical predictions of binding energy, carrier migration, and Fermi‐level alignment of V_2_O_5_/C_3_N_5_ heterojunctions were predicted and then experimentally synthesized. Z‐scheme transfer was verified in situ by XPS and radical trapping, with optimized samples evolving H_2_ at rates of 329.9 µmol h^−1^ and 7.21% AQE [237]. The heterojunctions between Ag_3_PO_4_ /MIL‐88A(Fe) and tetracycline were combined with Z‐system photocatalysis and photo‐Fenton degradation. Pathway and low toxicity were validated by adjusting Fe^2+^/Fe^3+^ ratios, whereas XPS, ESR, LC–MS, and QSAR supported the activity [238]. Zn_0.6_Cd_0.4_S/ZnO/g‐C_3_N_4_ demonstrated effective degradation of dye and antibiotics (98%–99%), and calculations and radical capture confirmed the dominance of the ·OH and ·O_2_ resulting [239].

Facet‐engineered Br‐doped Cu_2_O at NiFe‐LDHs enhanced the catechol degradation (95.7%), which was enhanced by the strength of internal fields and bulk charge separation [240]. In the case of reducing CO_2_, the reduction of CO and CH_4_ was 197.8 µmol h^−1^ and 130.3 µmol h^−1^ with B‐doped, *N*‐deficient g‐C_3_N_4_ coupled with ultrathin CoPc (CoPc/P‐BNDCN) using defect‐modulated bands and efficient Z‐scheme transfer [241]. The selective mechanism by which CN/CQD/CdZnS heterojunctions cleaved the 36.3 mg/g vanillin and vanillic acid was selective in cleaving the 36.3 mg/g 3‐O‐4 bonds of lignin, accelerated by CQDs [242].

The g‐C_3_N_4_/TS‐1 based on zeolites was, in turn, able to degrade Cr(VI) (99.9% in 5 min) or tetracycline (80% in 15 min) with enhanced light absorption and the formation of ·O_2_^−^ [243]. Ag_2_MoO_4_/BiOBr obtained 93.8% of methylene blue degradation through Z‐scheme carrier transfer mechanism [244]. Heterojunctions of TiO_2_ and Ga_2_O_3_ stimulated the CO_2_ reduction reaction by 2.5 and 9.6 times, respectively [245]. Ag_3_PO_4_/1T@2H‐MoS_2_ allowed the fast degradation of tetracycline (98.9% in 10 min) by suppressing electron–hole recombination [246]. FWO/BiOCl was dual CO_2_ reduced (CH_4_: 4.25, CO: 9.41 µmol g^−1 ^h^−1^) and bisphenol A degraded, which was further abetted with piezoelectric polarization and oxidants [247]. Together, by combining experiments and theory, correct band alignment, carrier transfer, and reactive species generation can be carefully controlled, which will hasten the design of Z‐scheme photocatalysts to convert solar energy and clean up the environment.

## 7 Performance Evaluation Metrics and Standards

### 7.1 Apparent Quantum Efficiency (AQE) and Solar‐to‐Hydrogen (STH) Efficiency

ZSHJs can be used to split water efficiently by utilizing solar energy but can typically be constrained by carrier recombination. Anatase TiO_2_/g‐C_3_N_4_ heterojunctions were hydrothermally synthesized, in which the g‐C_3_N_4_ was used as a template of anatase TiO_2_ enhanced carrier separation and H_2_ production. Redox mediators coupled with coupling WO_3_ or BiVO_4_ and β‐Ni(OH)_2_ produced AQEs of 4.94% (365 nm) and 4.01% (405 nm) [248]. Recombination was suppressed in a MgTa_2_O_6_ xNy/TaON heterostructure by spatial separation and defect elimination, with 6.8% AQE at 420 nm, an order of magnitude higher than both Pt‐TaON and all previous reported powdered Z‐schemes photocatalysts [249].

There is promise as well in QD‐based systems. Zn–AgIn_5_S_8_/α‐Fe_2_O_3_ recorded 1.7 mmol g^−1 ^h^−1^ H_2_ at 450 nm with 7.48% AQE assisted by electrochemiluminescence (ECL), TRPL, and ESR [250]. CdSe/ZnSe heterojunctions gave 15.3 mmol g^−1 ^h^−1^ H_2_ with 4.0% AQE at 400 nm [251], and AgBr/HCCN composites improved charge transfer, giving 6% AQE at 410 nm [252]. A ternary system with interfacial bonded‐induced electric fields (CdSe/SL‐ZnIn_2_S_4_/MoSe_2_) provided 70,789 µmol g^−1 ^h^−1^ H_2_ and 44.1% AQE at 420 nm [253]. First‐principles investigations of GeS/GeSe, SnC/WS_2_, and PtS_2_/g‐C_3_N_4_ heterostructures showed the optimal band alignment and interfacial charge redistribution, which allowed the achievement of 31–42 STH efficiencies [254, 255, 256]. STH efficiencies of 32.95% were obtained with 2D systems (SiSe/SnSe_2_, SiSe/SnSSe, AuSe/SnSe/SnS), and tensile strain and interlayer engineering increased the separation and kinetics [257, 258, 259]. This way, Z‐scheme photocatalysts have developed interface engineering, defect modulation, and dimensional control that have enabled record AQEs and high potential STH efficiencies of sustainable hydrogen generation.

### 7.2 Product Selectivity in CO_2_ Reduction and Organic Synthesis

The charge separation, transfer, and product selectivity of g‐C_3_N_4_/CuInS_2_ in CO_2_ reduction and organic synthesis were significantly higher in two‐metal active site g‐C_3_N_4_/CuInS_2_, which produced 246.75 µmol g^−1 ^h^−1^ CO with a selectivity of over 93% compared to H_2_ and simultaneous 100% inhibition of H_2_ by staggered band alignment [260]. g‐C_3_N_4_/FeWO_4_, a direct Z‐scheme, yields 6 µmol g^−1 ^h^−1^ CO with a 100% selectivity and 90% of suppression of H_2_ due to staggered band alignment [261]. Under AM 1.5G, 2D/2D CsPbBr_3_/Bi_2_WO_6_ gave 324 µmol g^−1 ^h^−1^ electron consumption and 1582 µmol g^−1^ product yield to produce CO (11.4%) and CH_4_ (84.3) [262]. Lead‐free Cs_2_ AgBiBr_6_ @g‐C_3_N_4_ was found to maintain steady CH_4_ generation through synergistic CB/VB orientation [263]. CH_4_ was produced by AgCl@ g‐C_3_N_4_ (53.03 µmol g^−1^), acetic acid (6.01 µmol g^−1^), and formic acid (2.51 µmol g^−1^) with no mediators [264]. ESR and DFT confirmed that ZnIn_2_S_4_/BiVO_4_ was an efficient CO_2_ reducer via direct Z‐scheme transfer [265].

CdS/g‐C_3_N_4_ was used in gram‐scale organic synthesis to couple benzylamine (199.79 µmol, 90 conversion) to C–N [266]. BiOBr/TzDa COF obtained 97% RhB and Cr(VI) removal [267], and BiOCl/g‐C_3_N_4_ transformed benzylamine with a yield of 96.1 and a selectivity of 95.2 [268]. All‐organic HOFs/g‐C_3_N_4_ provided H_2_ evolution (4450 µmol g^−1 ^h^−1^, AQE 22% at 450 nm) and total MO degradation [269]. CsPbBr_3_/Au/g‐C_3_N_4_ was used to oxidize C–H using Au as a mediator [270]. The combination of rational design of dimensionality, interfacial engineering, and band regulation in Z‐scheme photocatalysts allows selectivity in products to CO_2_ reduction and various organic transformations.

### 7.3 Stability and Recyclability Test

Photocatalytic heterojunctions can be practically applied as efficient catalysts if these remain stable with time, meaning they should maintain their structural integrity, retain their catalytic activity and show chemical stability during operation. The recyclability studies offer critical data to evaluate catalyst durability. In addition, structural characterization after the reaction with leaching analysis could distinguish true stability from deceptively retained activity. For instance, a ternary heterojunction of FeNi_2_S_4_@MoS_2_/g‐C_3_N_4_ exhibited excellent photocatalytic performance over five cycles of successive use, accompanied by negligible leaching of metals, signifying that the merged structure can retain functionality upon repeated cycles [271]. Similarly, the MCS/AIS photocatalyst exhibited high activity even after five degradation cycles, with no variation in morphology and crystalline structure after scanning electron microscopy (SEM) and XRD characterizations. The inductively coupled plasma mass spectrometry measurements also showed that there is virtually no leaching of Mn, In, and Ag, which is further evidence of chemical as well as structural stability [272].

Strategies for immobilization can improve recovery and resale value of a catalyst. The Ag–ZnO nanoflowers trapped within porous cellulose gel beads sustained stable performance during successive continuous flow operations. The low release of Ag^+^ and Zn^2+^ concentrations into the treated water suggests effective inhibition of secondary metals contamination. These immobilized architectures tackle significant practical challenges related to the issue of nanoparticle aggregation and the issue of catalyst separation after treatment. Similar stability is observed for CoFe_2_O_4_/Na_2_Ti_3_O_7_, which has been shown to withstand activity degradation over cycles of photocatalytic alcohol oxidation, although it suffers from a lack of quantitative leaching data, and most results evidence performance retention only [273, 274].

Long‐term photoelectrochemical testing serves as a more rigorous stability assessment. The fabricated ZnFe_2_O_4_/ZnIn_2_S_4_/NiCoP did not lose their activity during the chronoamperometric test for a long time. XRD, SEM, and elemental mapping of this material after testing showed that the as‐fabricated material retains its crystal structure and morphology after testing. Product examination further demonstrated the occurrence of elementary sulfur through the process of photo‐electrochemical sulfur oxidation, while repeated syntheses and stability measurements revealed good batch‐to‐batch reproducibility [275]. In a different system, the structural stability of NiS/PHI‐4 in saline conditions was due to the maintenance of the PHI structure and the suppression of alkali metal loss via cation exchange. Post‐reaction microscopy and diffraction analyses further supported the retention of the catalyst structure, although slight dissolution of NiS was observed [276].

In the case of heterogeneous photocatalysts, stability should also mean more than just retaining a high pollutant‐removal efficiency. The structure of the photocatalyst after recycling indicates that the active heterojunction is retained; metal leaching measurements are particularly critical for having potentially mobile metal components. Previous studies also showed that recycled heterojunction photocatalysts could maintain high degradation efficiencies with little detectable change in their XRD patterns, indicating good structural stability during repeated operation [277]. Similarly, the enhanced charge carrier transport in ternary Ti_3_C_2_ MXene/NiFe_2_O_4_/VO heterostructures due to closely spaced interfaces is also shown. However, morphological proof alone should not be taken to infer long‐term photocatalytic stability [278].

Recent S‐scheme and Z‐scheme systems further show that interfacial architecture can affect operational stability. A piezo‐photocatalyst constructed of Bi_4_Ti_3_O_12_@PDA/AgIO_3_ showed that fast charge transfer of the interfacial electrons and the carrier transfer role of the PDA layer enabled efficient separation of carriers and sustained multifunctional activity. However, these observations relate to charge transfer kinetics rather than recyclability per se [279]. The Cd/CdS/g‐C_3_N_4_ complex manifested continued performance in environmental remediation through synergy involving both Z‐scheme and Ohmic junction effects. However, mechanistic evidence for charge transfer must be differentiated from direct evidence for long‐term catalyst stability [280].

## 8 Applications of Z‐Scheme Photocatalysts

### 8.1 Overall Water Splitting and Hydrogen Production

ZSHJs can be used to produce H_2_ under sunlight irradiation as they are capable of sustaining high redox potentials with the effective separation of charges. Recombination was hindered by TiO_2_/g‐C_3_N_4_ hydrothermal synthesis to AQEs of 4.94% (365 nm) and 4.01% (405 nm). Redox mediators (I^−^/IO_3_^−^, Fe^2+^/Fe^3+^) are coupled with WO_3_ or BiVO_4_ to allow overall splitting of water, further enhanced by loading with 2D/3D Ni(OH)_2_ [281]. Defects are suppressed in (oxy)nitride MgTa_2_O_6_ Ny/TaON Z‐schemes using Pt‐loaded MgTa_2_ O_6_ as H_2_ and PtO_8_‐WO_3_ as O_2_ photocatalysts. They give 6.8% of AQE at 420 nm, or approximately 360 times that of single components with IO_3_^−^/I_3_‐mediators [249].

Design is also developed in 2D vdW systems. GaSe/ZrS_2_ is high mobility (approximately 1450 cm^2^ V^−1 ^s^−1^), high absorption (higher than 105 cm^−1^), and strain‐tuned (spontaneous) water splitting efficiency [282]. Direct Z‐schemes with MoSe_2_/SnS_2_ and C_3_ N/WS_2_ also obtain an efficient separation, high redox potential, and a broad pH range [283, 284]. Hybrid and core shell designs are performance‐enhancing designs. Until now, BiFeO_3_(TpPa‐1‐COF) synthesizes polarized fields and generates H_2_ and O_2_ rates of 1416.4 and 708.2 µmol g^−1 ^h^−1^ [285]. With no Pt cocatalysts, CoFe_2_O_4_ at ZnIn_2_S_4_ p–n heterojunctions achieve 1576.5 µmol g^−1 ^h^−1^ of H_2_ [286].

Noble metal mediation is also beneficial to activity. g‐C_3_N_4_/ZnO/Au attains 46.46 µmol g^−1 ^h^−1^H_2_ (approximately 25–30 times higher than g‐C_3_N_4_), with Au acting as the mediator and increasing absorption rate through LSPR [287]. B‐doped C_3_N_4_/ZnO demonstrates Z‐schemes‐to‐type‐II switching with 2.9‐fold enhancement in H_2_ with Fermi level tuning [288]. Heterostructures of other types, such as α‐Bi_2_O_3_/Cd_0.5_Zn_0.5_S and Mn_0.8_Cd_0.2_S/NiCo_2_S_4_, also provide high activity through intimate interfaces, with Mn_0.8_Cd_0.2_S/NiCo_2_S_4_ operating at 21.41 µmol g^−1 ^h^−1^ of H_2_ evolution in the absence of noble metals [288, 289]. CN/BCN heterojunctions of metal‐free 2D type yield H_2_ evolution rate of 3357.1 µmol g^−1 ^h^−1^ with 16.3% AQE at 420 nm and multiple stable performance of 15 cycles [290]. All these developments in 2D vdW interfaces, p–n junctions, noble metal decoration, and metal‐free systems allow Z‐schemes to become scalable systems to efficiently produce H_2_ and convert renewable energy into useful energy.

### 8.2 Photocatalytic CO_2_ Reduction to Fuels

ZSHJs have been identified to be very useful in the separation of charge carriers and preservation of large redox potentials, which allows conversion of CO_2_ to fuels selectively. As an example, 2D nanosheets and 1D nanobelts form a hybrid 3D ZnIn_2_S_4_/TiO_2_ system that produced CH_4_ at a rate of 1.135 µmol g^−1 ^h^−1^, 39 times higher than pure ZnIn_2_S_4_ because of direct transfer through the Z‐scheme [291]. On the same note, the Au‐mediated ZnCdS/g‐C_3_N_4_ systems produced CH_3_OH at 1.31 µmol g^−1 ^h^−1^, and the interfacial charge transport values were 43.6 and 32.7 times higher than that of pure ZnCdS and g‐C_3_N_4_, respectively [23]. Heterostructures of Bi_2_WO_6_/ZnIn_2_S_4_ produced 19.54 µmol g^−1 ^h^−1^ CH_4_, greater than single components because of improved CO_2_ adsorption and charge transfer [292]. Z‐scheme charge separation increases CO_2_ conversion efficiency, which is also indicated in Ag_3_PO_4_/g‐C_3_N_4_ systems, which yielded approximately 57.5 µmol g^−1 ^h^−1^ at 610 times the rate of g‐C_3_N_4_ and P25 [293]. Similarly, ZnFe_2_O_4_ /AgTiO_2_ nanorod heterojunctions enhanced the generation of CO, CH_4_, and CH_3_OH and 1D nanorods served as the reservoirs of charge [294]. More sophisticated 2D vdW heterostructures were also promising: C_2_N/aza‐CMP bilayers with Cu‐dimer anchoring reduced CO_2_ overpotential, and g‐C_3_N 4/SnS_2_ QD junctions bound CH_4_ selectively [295]. Other high‐performance systems are also based on WO_4_, g‐C_3_N_4_ nanosheets, which gave 4.57 µmol g^−1 ^h^−1^ of CH_4_ and 5.19 µmol g^−1 ^h^−1^ of CO with stable recrystallization [296], and CuO‐modified WO_3_ heterojunctions (15 wt% CuO) that gave high light absorption, charge separation, and hydrocarbon yields [297]. Altogether, Z‐scheme photocatalysts, such as 3D/2D hybrids, ternary composites, vdW heterostructures, and doped systems, show better performance in CO_2_ reduction, enhancing activity, selectivity, and stability to convert solar energy to fuels and to clean up the environment, as shown in Table 6.

**TABLE 6:** Summary of ZSHJ photocatalysts for photocatalytic CO_2_ reduction to fuels.

Photocatalyst system	Structural/functional features	Main products and performance	Reference
3D ZnIn_2_S_4_/TiO_2_ nanobelts	Direct Z‐scheme, 2D–1D hybrid with large surface area, efficient charge separation	CH_4_: 1.135 μmol g^−1 ^h^−1^ (39× higher than ZnIn_2_S_4_)	[291]
Zn_0_._5_Cd_0_._5_S/Au@g‐C_3_N_4_ (ternary)	Au as electron mediator, enhanced visible‐light absorption, and surface area	CH_3_OH: 1.31 μmol g^−1 ^h^−1^ (43.6× vs. ZCS, 32.7× vs. g‐C_3_N_4_)	[23]
3D Bi_2_WO_6_/ZnIn_2_S_4_	Electrostatic self‐assembly, abundant active sites, efficient CO_2_ adsorption	CH_4_: 19.54 μmol g^−1^ (4 h, simulated sunlight); 8.73× versus ZnIn_2_S_4_	[292]
Ag_3_PO_4_/g‐C_3_N_4_	In situ deposition, Z‐scheme charge separation	CO_2_ conversion: 57.5 μmol h^−1^ g^−1^ (6.1× vs. g‐C_3_N_4_, 10.4× vs. P25)	[293]
ZnFe_2_O_4_ microspheres/Ag–TiO_2_ nanorods	Indirect Z‐scheme, 1D TiO_2_ facilitates transport, enhanced visible absorption	CO, CH_4_, CH_3_OH yields higher than ZnFe_2_O_4_/TiO_2_	[294]
C_2_N/aza‐CMP VDW heterobilayers	2D/2D direct Z‐scheme, spatial carrier separation; Cu‐dimer anchoring lowers overpotential	Selective CH_4_ formation; high solar‐to‐fuel efficiency	[295]
g‐C_3_N_4_/SnS_2_ QDs	In situ deposition; l‐cysteine enhances CO_2_ adsorption and IEF	Enhanced CO_2_ reduction; HCOOH intermediate detected (FTIR)	[298]
W_18_O_49_/g‐C_3_N_4_ urchin‐like	Tight interfacial contact; W^5+^ LSPR enables NIR activity	CO: 6.46, CH_4_: 3.97 μmol h^−1 ^g^−1^	[296]
2D/2D g‐C_3_N_4_/BiVO_4_ nanosheets	Direct Z‐scheme, ultrathin sheets with face‐to‐face contact	CH_4_: 4.57, CO: 5.19 μmol g^−1 ^h^−1^; high recyclability	[299]
Cu‐doped WO_*x*_	Cu doping (15 wt%) optimizes absorption and charge separation	High hydrocarbon yield; stable activity over cycles	[297]

### 8.3 Photocatalytic H_2_O_2_ Production and Pollutant Degradation

ZSHJs can be used to synthesize H_2_O_2_ and degrade pollutants well since they separate charges very well but retain redox activity. CoWO_4_/Bi_2_WO_6_ 3D hybrids enable visible light harvesting and dual H_2_O_2_ oxidation (O_2_ reduction and water oxidation) as well as single and combined antibiotic degradation [300]. The Ti_3_C_2_/g‐C_3_N_4_/BiOCl Schottky GX system produced H_2_O_2_ (1275.13 µM) with increased electron transport [301]. One sample of defect‐engineered TiO_2−*x*_/g‐C_3_N_4−*x*_ showed a H_2_O_2_ of 1131.6 µmol L^−1^ h^−1^ H_2_O_2_, some six times higher than the same pristine sample, due to the formation of active sites and light absorption by vacancies [302]. MOF‐derived ZnIn_2_S_4_/Bi_2_S_3_ generated 995 mmol of H_2_O_2_ and removed 95% of tetracycline, which was 44.5 times more than the individual compounds [303]. Dual S‐vacancy hollow Ag_2_CdS/ZnIn_2_S_4−*x*_ produced 11,837 µmol g^−1 ^h^−1^ H_2_O_2_ by spatial charge separation and selective two‐electron O_2_ reduction [304]. ZnO/g‐C_3_N_4_‐F.V. produced 5312 µmol L^−1^ H_2_O_2_ in 8 h, achieving 97.4% inactivation of bacteria and molds, with •OH and •O_2_^−^ radicals acting as the primary reactive species [305].

Ephedrine was degraded using AgBr/P‐g‐C_3_N_4_ in the presence of solar light, in which h^+^, O_2_^−^ were the active species [19]. By catalyzing the reaction of •OH, •O_2_^−^, and h^+^, NiMoO_4_/g‐C_3_N_4_ removed ciprofloxacin and malachite green by 90.8% and 98.8%, respectively, in half a dozen cycles [19]. BiOI/Ag@AgI containing iodine vacancies enhanced the tetracycline degradation and recyclability [306]. Bi‐ZFO/BMO fully oxidized dyes and antibiotics in 50–110 min by using the assistance of Bi SPR, oxygen vacancies, and Z‐scheme transfer [307]. The use of Ag_2_ CrO_4_‐GO composites demonstrated increased degradation of the methylene blue by 3.5 times compared to Ag_2_CrO_4_ because of the enhancement of the charge separation and photocorrosion inhibition [308]. SnNb_2_O_6_/ZnO 2D/2D interfaces exhibited 15 and four times greater rate constants in the breakdown of RhB and phenol than the conductors, respectively [309].

Overall, hierarchically designed, defect‐engineered, metal/semiconductor interface‐based, and 2D/2D, Z‐scheme photocatalysts are demonstrated as highly versatile systems in visible‐light‐driven H_2_O_2_ production and environmental cleanup in general, as shown in Figure 5a–d. In Figure 5a, sequential photocatalytic production of H_2_O_2_ and degradation of pollutants by a Cu‐based ZSHJ: diagrammatic representation of the sequence of charge transfer reactions of the reaction and the formation of reactive species [310], (b, c) LDH/CTF‐1 heterojunction photocatalytic degradation of tetracycline (TC) which exhibits mechanistic evidence of Z‐scheme charge transfer, generation of reactive species, and band alignment [311], (c) ZnO, SnIn_4_S_8_, and ZnO–SnIn_4_S_8_ ZSHJs hydrothermal synthesis to maximize photocatalytic H_2_O_2_ production and pollutant degradation [312], (d) represents gradual hydrothermal production of ZW/MCS heterojunction using Na_2_WO_4_ and Zn (NO_3_)_2_ as precursors, which are preferable in the production of H_2_O_2_ through photocatalysis and degradation of pollutants [313].

**FIGURE 5:**
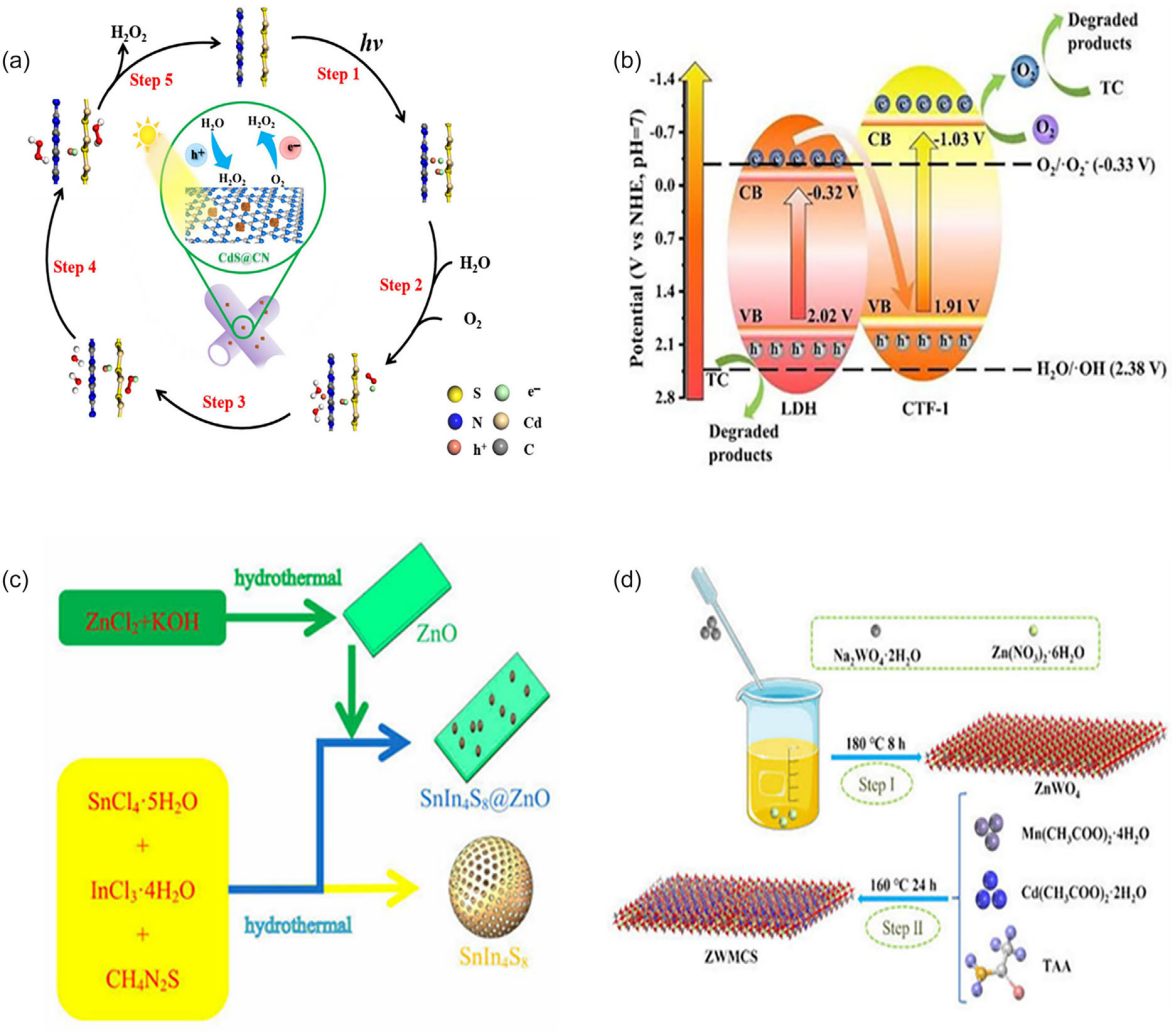
(a) Schematic illustration of the sequential photocatalytic process over the Cu‐based ZSHJ, showing H_2_O_2_ generation followed by pollutant degradation through charge transfer and the formation of reactive oxygen species [310]. (b) The photocatalytic degradation of tetracycline (TC) with LDH/CTF‐1 heterojunction showing the mechanistic evidence of Z‐scheme charge transfer mechanism, generation of reactive species, and band alignment [311]. (c) ZnO, SnIn_4_S_8_, Z + ZSHJ: Hydrothermal route to synthesize ZnO, SnIn_4_S_8_, and SnIn_4_S_8 _+ ZnO, to be used in maximizing the photocatalytic generation of H_2_O_2_ and the degradation of pollutants [312]. (d) Gradual hydrothermal preparation of ZW/MCS heterojunction by use of Na_2_WO_4_, Zn(NO_3_)_2_ as well as metal precursors, which is favorable to photocatalytic conversion of H_2_O_2_ and degradation of pollutants [313].

### 8.4 Solar‐Driven Organic Transformations and Green Chemistry

ZSHJs allow converting solar energy and green chemistry with energy and efficiency as they improve charge separation, redox capacity, and light harvesting. 1D/2D W_18_‐O_49_/g‐C_3_N_4_ performed better in the degradation of phenol and coupling of benzylamine, and ESR confirmed the presence of reactive species and direct transfer between the two in the Z‐scheme [314]. OER activity was enhanced through powerful interfacial interaction and subsequent Z‐scheme transfer in Ag_3_PO_4_/g‐C_3_N_4_ composites [11]. A bifunctional Z‐patterned H‐TiO_2_@ZIS@CoTPP‐S photocatalyst produced 158.15 µmol g^−1 ^h^−1^ CO, which was stimulated by the absorption of light energy, two electric fields, and the effective activation of CO_2_ [315]. All‐solid‐state composites of ZnIn_2_S_4_/CeO_2_ provided 664.1 µmol g^−1 ^h^−1^ benzaldehyde as well as 1496.6 µmol g^−1 ^h^−1^ of H_2_, selective toward alcohol oxidation [316]. Methylene blue was degraded by TiO_2_/Bi_2_WO_6_ photoelectrodes by a red‐shifted absorption and a productive Z‐Scheme transfer (94% in 160 min) [317]. ZnS/TiO_2_ nanowires with sulfur vacancies had 35.77 mmol g^−1^ of H_2_ and 89.1% benzyl alcohol oxidation to benzaldehyde [318]. CoTiO_3_/Cu‐BTC MOF heterojunctions eliminated Cr(VI) (98.3%) and degraded norfloxacin (97.5%), and •OH and •O_2_^−^ were the primary species [319]. Ternary LaFeO_3_/B‐CN/Fe_2_O_3_ degraded navy‐blue dye (98%) through synergistic Z‐scheme transfer and B‐doping (*E*_g _= 1.6 eV) [320]. *N*‐doped g‐C_3_N_4_/Nd‐ZnO resulted in an antimicrobial activity with over 95% MB and tetracycline removal [321].

AgI/FeVO_4_C_3_N_4_ with dual Z‐schemes Ag bridges allowed reduction of Cr(VI) almost to completion in 60 min by transfer via LSPR, enhancing the transfer [322]. WO_3_/ZnIn_2_S_4_ was found to degrade TBBPA (91.6) through •O_2_^−^ pathways, which was supported by ESR and LC–MS/MS [323]. Mesoporous (0D/1D) Fe_2_O_3_ @ AgVO_3_ showed 94.3% Cr(VI) photoreduction, 1.05 mV OER, and 6.2 mA cm^−2^ photocurrent in AM 1.5G condition [324]. Altogether, Z‐scheme photocatalysts can be applied to perform organic synthesis, degrade pollutants, and split water, and vacancy engineering, interface design, and ternary design are some of the strategies to further develop sustainable energy and environmental remediation.

## 9 Scalability, Integration, and Reactor Design

### 9.1 Immobilized Thin Films

Thin films are immobilized to address the drawbacks of powder photocatalysts (recovery, stability, recycle) and ZSHJs in addition to improving charge separation and redox capacity. A spin‐coated NiFe_2_O_4_/NiO film on stainless steel (sol–gel coating) reduced CO_2_ and produced H_2_, 95.78% Congo Red was degraded (180 min) and 381.87 µmol dm^−2^ of H_2_ was produced, and active life persisted after five cycles [325]. All‐solid‐state Z‐schemes of RhB and tetracycline degradation were made on glass with TiO_2_/g‐C_3_N_4_ films and were optimally active on five layers [59]. LaCoO_3_/La‐g‐C_3_N_4_ immobilized through polyaniline films reached 97.2% enrofloxacin degradation in 60 min, 91% COD, and 83.5% total organic carbon removal of wastewater; •O_2_^−^ and •OH were predominant species [326].

Films of g‐C_3_N_4_/FeO_8_/H_2_O_2_ (pulsed laser deposition with thermal synthesis) demonstrated petal‐like structures, which increased ciprofloxacin degradation rate to *k* = 8.28  × 10^−4 ^min^−1^ (photocatalysis) and 2.6 × 10^−3 ^min^−1^ (photo‐Fenton), respectively, compared to FeO_8_/H_2_O_2_ controls [59]. Films on Bi plates BiOBr/BiVO_4_ (electrochemical ion exchange) produced 95.27 mmol g^−1 ^h^−1^CO, which is higher than either of the components; DFT proved the Z‐scheme transfer [327]. CdS/Cd‐BiOCl films (15%) exhibited better tetracycline elimination Z‐scheme transfer using metallic Cd as the mediator; •O_2_^−^ and •OH were the primary species [328]. ZnO/ZnCo_2_O_4_/Co_3_O_4_ films on cobalt foil combined Z‐scheme (ZnO/ZnCo_2_O_4_) and Type II (ZnCo_2_O_4_/Co_3_O_4_) heterojunctions, with 80.7% norfloxacin degradation and H_2_ yield of 357.12 µmol dm^−2^ [329]. FeSiB/TiO_2_ hybrid films produced by painting had 98.2/85.5%, respectively, tetracycline degradation in 4 h at pH 2/12 and could be recycled three times [330]. Hence, the idea of immobilized Z‐schemes thin films allows to generate and efficiently degrade pollutants and produce solar fuels, and the interface design, immobilization tactics, and morphology optimization determine the performance.

### 9.2 Photoreactor Configurations and Scale‐up Challenges

Photocatalytic conversion of CO_2_ and water provides a sustainable way of producing fuels but has challenges such as poor yields, low stability, and price. ZSHJs enhance charge separation, stability, and scalability of variants of photoreactor designs. A continuous‐flow reactor utilized reduced titania Cu_2_O, Z‐schemes, which provided CO_2_ to CH_4_ reduction (462 nmol g^−1^, 0.13%) in 42 h by inhibiting the Cu_2_O photocorrosion process [331]. A ternary CuWO_4_/Bi_2_S_3_/ZIF_67_ dual Z‐scheme degraded metronidazole and cephalexin with efficiencies of 95.6% and 90.1% in batch and flow reactors with a stability of more than six cycles; OH radicals prevailed [332]. Ag/Ag_2_O‐BiVO_4_ films improved the degradation of dye approximately 28 times (*k*_obs_ > 0.45 min^−1^) under blue LEDs or sunlight despite the constraints of recombination [333].

g‐C_3_N_4_ catalysts were also studied in a gastight, 20 mL, stainless‐steel reactor, the isotopic controls indicated consistent reduction of CO_2_ with catalysts, and reactor design was noted to show reproducible catalysts [334]. V_2_AlC/ZnCo_2_O_4_/TiO_2_ monoliths were comparable to fixed‐bed, monolith, and flow reactor with selectivity control: fixed‐bed preferred CO/H_2_, and monolith preferred CH_4_/H_2_ with yields up to 49 313 mmol g^−1^ CO [335]. Pt/GaP–TiO_2_–SiO_2_:Rh (PGTSR) with a high defect density made it possible to run overall water and seawater separation, with HER/OER of 80.1/41.2 mmol^−1^, twin reactors with ion shuttle membranes continued to operate with H_2_/O_2_ separation [336]. CdS/g‐C_3_N_4_ nanorod‐QDs improved C‐N interactions to achieve cell concentrations of 199.79 µmol (4.3 higher than pristine g‐C_3_N_4_) and nearly 100% gram‐scale conversion in 100 h [266].

Mineralized levofloxacin scalable Np‐(3) BTO@CuO films imitated in helical plug flow reactor 120 min faster than batch systems, which was authenticated by CFD modeling and spectroscopy [337]. α‐Fe_2_O_3_/ZnO/rGO heterostructures in helical plug flow reactor are 2.3‐fold faster than batch systems, verified by CFD modeling and spectroscopy [338]. In general, both ZSHJs in built‐in reactor designs lead to better charge separation, selectivity, and stability. The geometry of the reactor, light distribution, and mass transfer are the critical parameters used to scale photocatalytic processes to an industrial scale.

### 9.3 Integration With Photoelectrochemical (PEC) Devices

ZSHJs with PEC systems increase charge separation and maintain good redox potentials and device stability, which consequently allows effective water splitting, pollutant degradation, and biosensing. Indicatively, as shown by a nano‐sized pn junction consisting of Cu_2_O‐based on ZnO nanorods and Au, an onset potential of −0.330 V and a photocurrent density of −0.206 mA cm^−1^ were obtained, which is a significant improvement on *n*‐Cu_2_O because of the synergistic coupling between internal electric fields and Z‐scheme charge transfer processes [339]. The IPCE of tandem PEC architectures, including flexible MoSe_2_/CdS/ZnO on Au paper, was 52% at 460 nm, with a photocurrent of 2.4 mA cm^−2^, and H_2_ evolution rate of 39.7 µmol cm^−2 ^h^−1^, which had been reported as highly efficient in PEC water splitting [340]. Other applications of ZSHJ other than energy conversion include PEC sensing and remediation. It was discovered that ternary systems of Bi/Bi_2_WO_6_/g‐C_3_N_4_ were 3.4–10.4 times more sensitive than the corresponding individual components, allowing the detection of chlorpyrifos at 3.3 pM [341]. CdTe/Cd/ZnO‐Sb arrays, which took advantage of the synergistic piezo/p–n field effects, generated a photocurrent of −3.86 mA cm^−2^, an H_2_ generation of 58.5 mol cm^−2^, and 84.7% Faradaic efficiency [342]. Likewise, TiO_2_/Ti_3_C_2_ T*_x_*/Cu_2_O heterostructures allowed self‐powered PEC glucose sensing with a detection limit of 100 nM [343].

To remediate the environment, multi‐heterojunction TiO_2_‐BiVO_4_‐BP/RP films broke down 96.5% of fluxapyroxad in 120 min with 45% defluorination and maintained its stability in 10 cycles [344]. BiVO_4_/WSe_2_ (BVTS) photoanodes were also effective at catalyzing ampicillin degradation [345], and CdS/ZnS (CSZS–VZn) heterostructures were used to detect microRNA‐122 (10 aM–100 pM, 3.3 aM LOD) [346]. Application 3D ZIS/WO_3_ heterostructures detected PSA with a limit of detection of 5 pg/mL [347], AgI/Ag/BiOI arrays detected chloramphenicol at 0.226 nM [348], and CdS‐NPs@ZnO‐NRs core shell removed 94.07% tetracycline in 90 min with a photocurrent of 3.002 mA cm^−2^ and an overpotential of 171 mV [349]. In general, the incorporation of ZSHJs into PEC devices increases light‐harvesting, interfacial charge transfer, and redox functions in scalable applications in clean energy synthesis, pollutant treatment, and biosensing, as a summary of Table 7.

**TABLE 7:** Summary of ZSHJ integration with PEC devices for energy and environmental applications.

Photocatalyst system	Application	Key features and outcomes	Reference
ZnO NRs/Au/p–n Cu_2_O	PEC water splitting	Onset potential −0.330 V; photocurrent density 0.206 mA cm^−2^; 237% enhancement due to p–n junction and Z‐scheme separation	[339]
MoSe_2_–CdS–ZnO on Au paper (tandem Z‐scheme)	Flexible PEC photoanode for H_2_ generation	IPCE 52% @ 460 nm; current density 2.4 mA cm^−2^ @ 0.3 V versus NHE; H_2_ yield 39.7 μmol cm^−2^ h^−1^	[340]
Bi/Bi_2_WO_6_/g‐C_3_N_4_	PEC aptasensor (chlorpyrifos)	Photocurrent 0.7 μA; detection limit 3.3 pM; linear 0.01–1000 nM; recovery 98.4%–102.7%	[341]
*n*‐CdTeCd/p‐ZnO_*x*_Sb nanorods (bi‐piezoelectric Z‐scheme)	PEC water splitting	Photocurrent −3.86 mA cm^−2^; H_2_ evolution 58.5 μmol cm^−2 ^h^−1^; Faradaic efficiency 84.7%	[342]
TiO_2_/Ti_3_C_2_T*_x_*/Cu_2_O	PEC glucose biosensing	Self‐powered sensor; detection limit 33.75 nM; linear 100 nM–10 μM; high selectivity	[343]
TiO_2_–BiVO_4_–BP/RP multi‐heterojunction films	PEC pollutant degradation	96.5% fluxapyroxad removal in 120 min; 45.41% defluorination; stable for 10 cycles	[344]
BiVO_4_/WSe_2_ (BVTS)	PEC degradation of pharmaceuticals	Enhanced interfacial charge transfer, ROS generation, and long‐term durability	[345]
CdS/ZnS with Zn vacancies (CSZS–VZn)	PEC biosensor (microRNA‐122)	Detection range 10 aM–100 pM; limit 3.3 aM; Exo III‐assisted amplification	[346]
3D ZnIn_2_S_4_/WO_3_	PEC immunosensor (PSA detection)	Linear range 0.01–500 ng/mL; detection limit 5 pg/mL; high sensitivity and reproducibility	[347]
3D AgI/Ag/BiOI arrays	PEC aptasensor (chloramphenicol)	Wide linear range 2–250 nM; detection limit 0.226 nM; biocatalytic precipitation amplification	[348]
CdS‐NPs@ZnO‐NRs (core–shell)	PEC tetracycline degradation and water splitting	94.07% degradation (90 min); photocurrent 3.002 mA cm^−2^; overpotential 171 mV @ 2 mA cm^−2^	[349]

## 10 Challenges and Future Perspectives

### 10.1 Mechanistic Ambiguities

ZSHJs have potential applications in the fields of pollutants degradation, water purification, CO oxidation, and selective chemical transformations, but the mechanisms of charge transfer, recombination, and oxidation are not understood. The dynamics are essential to understand in order to design rational photocatalysts. Average oxidation state and carbon oxidation state increased to 1.35 and 2.94, respectively, by using AgIn_5_S_8_/ZnO heterojunction to treat pharmaceutical wastewater under the influence of internal electric fields that facilitated the spatial separation of charges and high redox capability [350]. MoS_2_/g‐C_3_N_4_ adhered to the direct Z‐scheme behavior through band bending, but the transfer was low due to low tunneling probability; metallic layers (Ag, Al, Au, Pt) provided fast charge channels, and Al made effective Ohmic contacts as a cost‐effective substitute [351]. In BiOI/g‐C_3_N_4_, interface non‐radiative recombination centers enhanced the reduction of Cr(VI) (79.2% within 15 min), as shown by PL, PA, WF, and ESR studies [352]. Tenfold CO oxidation was observed in FePc/Ag/TiP nanosheets compared to bulk TiP and was enhanced compared to TiO_2_ (P25) under Ag‐assisted Z‐scheme transfer and directional O_2_ activation [353]. Co_3_O_4_/NiCo_2_O_4_ hetero‐nanocages containing VO_2_ cavities allowed the visible‐light CO_2_ methanation with a 96.3% CH_4_ selectivity and 20.32 µmol g^−1 ^h^−1^ carbon formation, with Ni atoms being able to tune Co, d‐band centers in order to influence atomic‐level charge control [354]. Heterojunctions of BiVO_4_ at ZnIn_2_S_4_ improved the production of hydrogen peroxide (1585.99 µmol g^−1 ^h^−1^) with a 2D/3D interaction and adsorption of O_2_ [355]. Photo‐piezocatalysis using the *N*‐vacancy g‐C_3_N_4_ homojunctions was reported, with type‐II and Z‐schemes varying, enhancing RhB degradation and H_2_O_2_ generation [356].

COF‐TaTp/SnS_2_ S‐schemes were found to have very high internal fields (2.14–4.63 times higher than as components) with C─N─S bonds selecting bands and hybrid states to generate visible NIR–H_2_O_2_ [357]. Ferroelectric polarization and S‐scheme transfer were used in KNbO_3_‐CuO, where the production of CH_4_ was increased by 26 times with a selectivity of 97.8% [358] and g‐C_3_N_4_‐CeO_2_ with persulfate (PDS), which oxidized bisphenol A (94.2% selectivity) nonradically [359]. Therefore, Z‐ and S‐scheme heterojunctions enhance photocatalysis, but the specific interfacial charge dynamics, recombination, and vacancy‐mediated processes remain unclear. Mechanistic understanding is essential in developing the new generation photocatalysts to energy and environmental use.

### 10.2 Long‐Term Stability and Short‐Term Stability

On solar energy conversion and environmental remediation, stabilizing Z‐scheme photocatalysts is necessary. There are strategies such as heterojunction engineering, modulation of defects, core–shell designs, and plasmonic integration [360]. ZnIn_2_S_4_/TiO_2−*x*_ heterojunctions used the sulfur‐Ti^3+^/oxygen vacancy interactions, which prolonged the light absorption and stabilized the sites. The best 30ZIS/TiO_2_ yielded H_2_ evolution more than 20‐ and fourfold better than TiO_2_ and ZnIn_2_S_4,_ with no loss of activity, as did g‐C_3_N_4_/ZnIn_2_S_4_ with 91.3% selectivity [361]. CuMn_2_O_4_/GO heterostructures increased RhB (84.46) and MB (99.82) degradation after 10 cycles. They also had a low overpotential (267.6 mV), Tafel slope (122.4 mV/dec), charge transfer resistance (2.13 Ω), and long‐term electrochemical stability [362]. Recombination and sulfide photocorrosion were inhibited in MoS_2_@ In_2_S_3_/Bi_2_S_3_ core–shell heterojunctions, with H_2_ evolution rates of 973.42 µmol h^−1^ halved and tetracycline degradation of 99.6% in 90 min [363]. ZnS/Fe_2_O_3_ nanohybrids allowed 95.6% RhB degradation in 60 min and complete *E. coli* inactivation, which was stable up to six cycles [364]. V_2_O_5_/β‐Bi_2_O_3_ employed thermal phase transition and incorporation of vanadium to trigger interfacial fields in obtaining >98% Hg^0^ elimination and 95% retention over 35 h [365]. Plasmonic Bi^0^ with Z‐scheme junctions of H_2_O_2_ (953 µmol g^−1 ^h^−1^) and NH_4_^+^ (131 µmol g^−1 ^h^−1^) was produced by CeVO_4_/Bi/Bi_2_MoO_6_ with the use of 4–20 times as much kinetics compared to binary systems [366]. The levofloxacin degradation capacity was maintained at 99%, 95%, and 92% in a ternary Z‐scheme after three consecutive cycles [367]. TiO_2_/ZnTe/Au nanocorncobs were under illumination to exploit the Au plasmonics to stoichiometrically split water (3344 μmol g^−1 ^h^−1^, 0.98% STH), and morphology was maintained under light [368]. Junctions, including g‐C_3_N_4_–Ag_3_PO_4_, Fe_2_(MoO_4_)_3_/Ag, and Ag_3_PO_4_, were photoresistant, redox potential stable, and could be used repeatedly with minimum degradation [369, 370]. Altogether, interfacial coupling, control of vacancies, plasmonic enhancement, and manipulation of heterostructure are essential to reach stability in Z‐scheme systems, active sites, recombination suppression, and preservation of structure during prolonged operation.

## 11 Conclusions

Heterojunction photocatalysts based on Z‐scheme have emerged as versatile platforms for coupling efficient charge separation with strong redox capability. They can pave the way for solar‐driven environmental remediation, hydrogen evolution, CO_2_ conversion, and value‐added chemical transformations. Photocatalytic benefits of Z‐scheme systems cannot be interpreted from either the band alignment or enhanced catalytic activity only. For a rigorous mechanistic understanding, correlation of the interfacial structure, electronic configuration, charge redistribution, carrier dynamics, and surface reaction chemistry is required. One cannot distinguish between type‐II, Z‐scheme, and S‐scheme pathways easily as similar photocatalytic responses arise from fundamentally different charge transfer processes.

According to the analysis, approaches, such as Mott–Schottky, VB–XPS, UV–vis spectroscopy, PL, photocurrent, electrochemical impedance spectroscopy, EPR, and radical trapping experiments, have provided evidence that is valuable but generally indirect. More dependable mechanistic assignments must take account of additional characterization, including work function measurements, KPFM, surface photovoltage spectroscopy, dark/light and in situ irradiated XPS, photodeposition, and ultrafast transient spectroscopy, DFT calculations where appropriate. Observations should be viewed in unison, and not any one measurement taken as proof of a Z‐ or S‐scheme mechanism. In addition, photocatalytic performance should be decoupled from intrinsic charge transfer effects since surface area, adsorption, defects, light harvesting, cocatalysts, and mass transfer processes can all independently affect apparent activity. Future studies should focus on long‐term stability, photocorrosion resistance, elemental and mediator leaching, maintenance of interfacial coupling, performance in real wastewater, and continuous‐flow conditions for practical applications. Essential aspects also include standardized activity reporting, quantitative characterization of interfacial electric fields, mineralization and toxicity assessment, immobilized catalyst architectures, scale‐up, techno‐economic evaluation, and life‐cycle assessment. The future design of Z‐scheme photocatalysts should not only rely on the experimental optimization of materials but also aim at interface engineering that is experimentally verified, quantitatively characterized, and application‐oriented. This will give a more reliable path from fundamental charge transfer concepts to sustainable solar energy and sustainable environmental technologies.

## Author Contributions

**Mahalakshmi** **and** **Radhakrishnan:** conceptualization, methodology, data curation, writing – original draft. **Manikandan Ayyar** **and** **Subasree:** software, resources, visualization. **Zeinebou Yahya:** writing – review and editing, project administration, supervision, validation. **Lalitha Gnanasekaran** **and** **Shanmugapriya:** writing – review and editing, resources, methodology. **Abinet Gosaye Ayanie:** former analysis, validation, writing – review and editing.

## Funding

The authors have nothing to report.

## Conflicts of Interest

The authors declare no conflicts of interest.

## Data Availability

The data that support the findings of this study are available from the corresponding author upon reasonable request.
